# Micro-Meta App: an interactive tool for collecting microscopy metadata based on community specifications

**DOI:** 10.1038/s41592-021-01315-z

**Published:** 2021-12-03

**Authors:** Alessandro Rigano, Shannon Ehmsen, Serkan Utku Öztürk, Joel Ryan, Alexander Balashov, Mathias Hammer, Koray Kirli, Ulrike Boehm, Claire M. Brown, Karl Bellve, James J. Chambers, Andrea Cosolo, Robert A. Coleman, Orestis Faklaris, Kevin E. Fogarty, Thomas Guilbert, Anna B. Hamacher, Michelle S. Itano, Daniel P. Keeley, Susanne Kunis, Judith Lacoste, Alex Laude, Willa Y. Ma, Marco Marcello, Paula Montero-Llopis, Glyn Nelson, Roland Nitschke, Jaime A. Pimentel, Stefanie Weidtkamp-Peters, Peter J. Park, Burak H. Alver, David Grunwald, Caterina Strambio-De-Castillia

**Affiliations:** 1 Program in Molecular Medicine, UMass Chan Medical School, Worcester, MA USA; 2 Department of Biomedical Informatics, Harvard Medical School, Boston, MA USA; 3 Advanced BioImaging Facility (ABIF), McGill University, Montreal, Quebec Canada; 4 RNA Therapeutics Institute, UMass Chan Medical School, Worcester, MA USA; 5 Janelia Research Campus, Howard Hughes Medical Institute, Ashburn, VA USA; 6 Institute for Applied Life Sciences, University of Massachusetts, Amherst, MA USA; 7 Department of Anatomy and Structural Biology, Gruss-Lipper Biophotonics Center, Albert Einstein College of Medicine, Bronx, NY USA; 8 BioCampus Montpellier (BCM), University of Montpellier, CNRS, INSERM, Montpellier, France; 9 Institut Cochin, Inserm U1016-CNRS UMR8104-Université de Paris, Paris, France; 10 Center for Advanced Imaging, Heinrich-Heine University Duesseldorf, Düsseldorf, Germany; 11 UNC Neuroscience Microscopy Core Facility, Department of Cell Biology and Physiology, Carolina Institute for Developmental Disabilities, and UNC Neuroscience Center, University of North Carolina, Chapel Hill, NC USA; 12 Department of Biology/Chemistry and Center for Cellular Nanoanalytics, University Osnabrück, Osnabrück, Germany; 13 MIA Cellavie Inc., Montreal, Quebec Canada; 14 Bioimaging Unit, Newcastle University, Newcastle upon Tyne, UK; 15 UNC Neuroscience Microscopy Core Facility, Carolina Institute for Developmental Disabilities, and UNC Neuroscience Center, University of North Carolina, Chapel Hill, NC USA; 16 Center for Cell Imaging, University of Liverpool, Liverpool, UK; 17 Microscopy Resources of the North Quad, University of Harvard Medical School, Boston, MA USA; 18 Life Imaging Center and Signalling Research Centres CIBSS and BIOSS, University of Freiburg, Freiburg, Germany; 19 Laboratorio Nacional de Microscopía Avanzada, Instituto de Biotecnología, Universidad Nacional Autónoma de México, Cuernavaca, Mexico

**Keywords:** Confocal microscopy, Data publication and archiving, Standards, Software, Wide-field fluorescence microscopy

## Abstract

For quality, interpretation, reproducibility and sharing value, microscopy images should be accompanied by detailed descriptions of the conditions that were used to produce them. Micro-Meta App is an intuitive, highly interoperable, open-source software tool that was developed in the context of the 4D Nucleome (4DN) consortium and is designed to facilitate the extraction and collection of relevant microscopy metadata as specified by the recent 4DN-BINA-OME tiered-system of Microscopy Metadata specifications. In addition to substantially lowering the burden of quality assurance, the visual nature of Micro-Meta App makes it particularly suited for training purposes.

## Abstract (web summary)

Micro-Meta App is an intuitive, highly interoperable, open-source software tool designed to facilitate the extraction and collection of relevant microscopy metadata as specified by recent community guidelines.

## Main

For microscopy images to be appropriately interpreted and reproduced, and to satisfying Findable Accessible Interoperable and Reusable (FAIR) principles^1^, they should be accompanied by detailed descriptions of microscope hardware, image acquisition settings, image pixel and dimensional structure and instrument performance. Currently, documentation of imaging experiments is seriously impaired by the lack of easy-to-use software tools that facilitate the extraction and collection of relevant microscopy metadata. Micro-Meta App is an intuitive open-source software designed to tackle these issues that has been developed in the context of the 4D Nucleome (4DN) consortium^2,3^, and of nascent global bioimaging community organizations, including BioImaging North America (BINA)^4,5^ and Quality Assessment and Reproducibility in light microscopy (QUAREP-LiMi)^6–8^, whose goal is to improve reproducibility, data quality and sharing value for imaging experiments. The App provides a visual interface for building comprehensive descriptions of the conditions used to produce microscopy datasets as specified by the 4DN-BINA-OME tiered-system of Microscopy Metadata specifications^9–13^. To ensure wide adoption by microscope users with different skill levels and needs, Micro-Meta App is ideally suited for training purposes and interoperates closely with MethodsJ2 (refs. ^14,15^) and OMERO.mde^16,17^, two complementary tools described in parallel manuscripts.

### Microscopy metadata improve data quality and reproducibility

In addition to providing essential information about the provenance (that is, origin, lineage)^18,19^ of microscopy results, the establishment of community-driven, documentation and quality control (QC) specifications for light microscopy would make it possible to faithfully interpret scientific claims, facilitate comparisons within and between experiments, foster reproducibility and maximize the likelihood of data reuse^6–8,20–25^. First and foremost, such information would facilitate the compilation of accurate Methods sections for scientific publications^26–29^. Furthermore, it would provide clear guidance to microscope and software manufacturers about what information should be written automatically in the headers of image files during image acquisition to ensure scientific rigor. Finally, machine-actionable versions of the same information^30^ could be uploaded alongside image-datasets on the growing number of public image data resources^22,31–43^ that allow the deposition of raw image data associated with scientific manuscripts, emulating for light microscopy the successful path that led to genomics community standards^44–48^.

To promote the development of shared standards, the NIH-funded 4DN^2,3^ and the CZI-funded BINA^4,5^ have recently proposed the 4DN-BINA-OME (NBO) Microscopy Metadata specifications^9–13^. These specifications consist of an extension of the Open Microscopy Environment (OME) data model (that is, the basis for the widely adopted BioFormats library)^49–52^, which is organized in three tiers (details in Supplementary Information), and allows the classification of imaging experiments into levels of increasing complexity^11–13,53^. These specifications not only provide an OME-based comprehensive set of metadata that should be recorded, but they also specify which information subset should be included depending on experimental intent, technical intricacy and image analysis needs. The 4DN-BINA-OME specifications lay the foundations for upcoming community-sanctioned standards being developed by QUAREP-LiMi^6,7^. Their purpose is to provide a scalable, interoperable and OME-Next-Generation File Format (NGFF)^54,55^ compatible framework, guiding scientists as to what provenance metadata and calibration metrics should be afforded to ensure quality, reproducibility and value for different categories of light microscopy experiments.

**Fig. 1:**
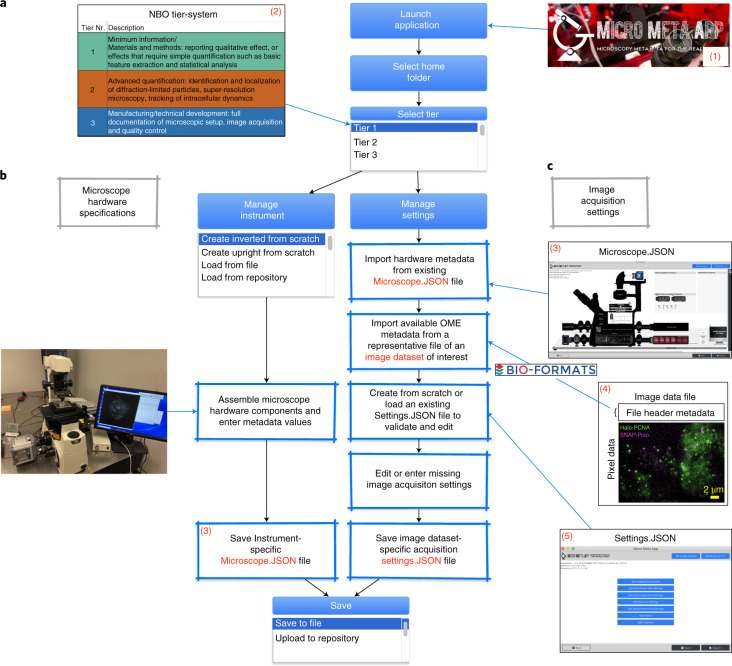
Micro-Meta App data processing workflows. Flowchart depicting the three sections of Micro-Meta App (1). **a**, The function of the initial section is to select among the three available 4DN-BINA-OME^11,13^ documentation tier levels (2), which is most appropriate for a given experimental -design, instrument-complexity and image analysis needs (details in Supplementary Information). **b**, The purpose of the *Manage Instrument* section is to create (or edit) a visual representation of the hardware configuration of a given microscope, which is then saved in a Microscope.JSON file (3) containing a list of components and associated 4DN-OME-BINA-specified metadata-information. **c**, The aim of the *Manage Settings* section is to collect relevant hardware information from an existing Microscope.JSON file (3), use BioFormats^52^ to retrieve existing OME-compatible microscopy metadata from an image of interest (4) and create (or edit) a Settings.JSON file (5) containing a rigorous description of the acquisition settings used for a given imaging experiment.

**Fig. 2:**
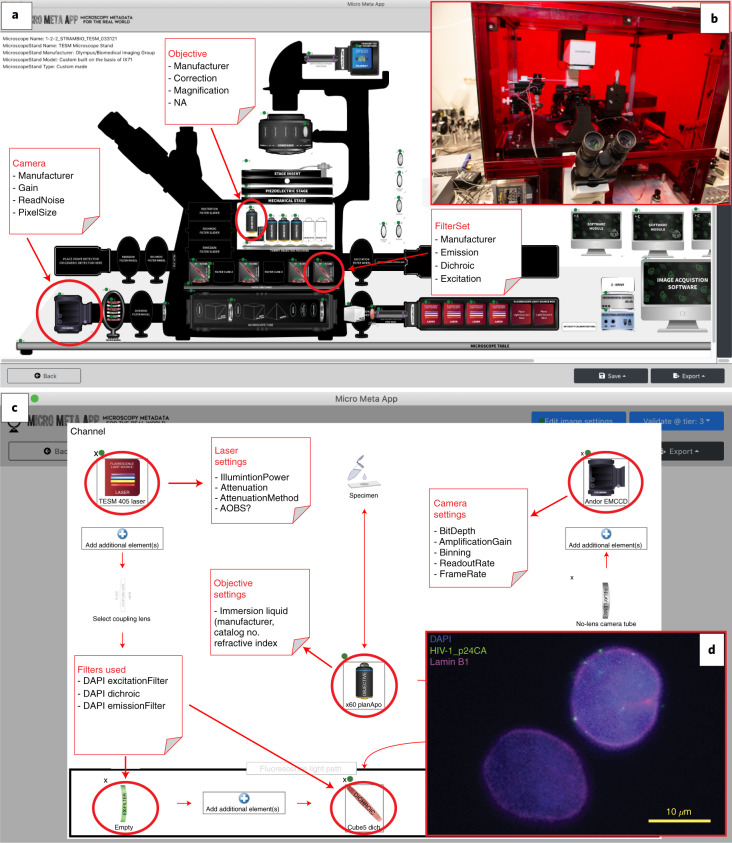
Micro-Meta App allows the intuitive and interactive documentation of imaging experiments. **a**, Illustrated is the use of the Manage Instrument section of Micro-Meta App to build a graphical record of the hardware components comprising the TIRF Epifluorescence Structured light Microscope (TESM) that was custom built at the Biomedical Imaging Group of UMass Chan Medical School. The resulting diagram allows the collection of all metadata-information sanctioned by Tier 3 (Fig. 1 and Supplementary Information) of the 4DN-BINA-OME model^11,13^. Specifically, by clicking on icons representing individual hardware components (for example, Objective, Filter Set or Camera), it is possible to record specific information describing each of them (for example, Objective Manufacturer, Model, Magnification and Numerical Aperture (NA)). **b**, Photograph depicting the TESM microscope illustrated in **a**. **c**, Use of the Manage Settings section of Micro-Meta App to build a graphical record of the lightpath (that is, from the illumination source to the detector) used for the acquisition of the 4,6-diamidino-2-phenylindole (DAPI) channel of the image in **d**. As shown, clicking on icons positioned along the lightpath it is possible to record the acquisition parameters (for example, Camera Bit Depth, Amplification Gain, Binning, etc.) that were used during the acquisition of a given image. **d**, Representative image obtained using the indicated microscope and settings (**a** and **c**).

**Extended Data Fig. 1:**
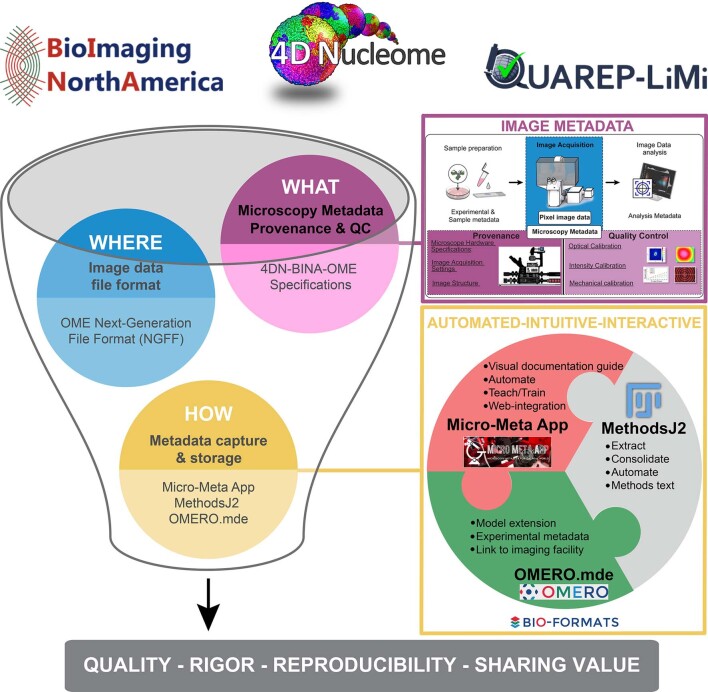
Quality, rigor, reproducibility and sharing value for imaging experiments require the definition of community-driven Microscopy Metadata specifications and the adoption of easy-to-use metadata collection tools to facilitate the documentation and quality control tasks for experimental scientists. The establishment of FAIR^1^, community-driven Microscopy image data Standards implies parallel development on three interrelated fronts: 1) WHERE: Next-Generation File Formats (NGFF) where the ever-increasing scale and complexity of image data and metadata would be contained for exchange^55^ (*blue bubble*). 2) WHAT: Community-driven specifications for what ‘data provenance’ information (microscope hardware specifications, image acquisition settings and image structure metadata) and quality control metrics are essential for rigor, reproducibility, and reuse and should therefore be captured in Microscopy Metadata (*magenta bubble*). 3) HOW: Shared rules for how the (ideally) automated capture, representation and storage of Microscopy Metadata should be implemented in practice (*yellow bubble*). Micro-Meta App, MethodsJ2 and OMERO.mde^14–17,63^ are three highly interoperable tools and complementary that function to: 1) train users on the importance of documentation and quality control; 2) facilitate metadata extraction, collection, and storage; 3) automatically write Methods sections; and 4) facilitate the development of experimental metadata specifications in connection with local core facilities. The different tools leverage different use- styles and preferences and are based on different software platforms in order to appeal to the broadest community including microscope builders, imaging scientists working in core facilities and experimental scientists. Thus: Micro-Meta App is used as a stand- alone app and can be integrated in third-party data portals^2,59,60,62^. MethodsJ2 works as an ImageJ plugin. OMERO.mde works in the context of the OMERO image data repository. The concept is to bring the tools to software platforms people are already using and lower the bar to enable broad uptake.

To render metadata specifications and QC standards actionable and easy to adopt, experimental scientists require software tools (or, even better, automated pipelines) to easily extract all available metadata from microscope configuration and image files and produce well-documented, high-quality, reproducible and reusable datasets. Despite some advances^26,56,57^, current tools offer limited functionalities, and are not integrated with community standards. Here, we present a suite of three interoperable software tools (Extended Data Fig. 1) that were developed to provide complementary, intuitive approaches for the bench-side collection of experimental and microscopy metadata^10,11,13^. In two related manuscripts, we describe: (1) OMERO.mde, which emphasizes the development of flexible, nascent specifications for experimental metadata^16,17,58^ and (2) the ImageJ/Fiji MethodsJ2 plugin^14,15^, which automatically generates Methods for scientific publications. Here, we present Micro-Meta App (Figs. 1 and 2 and Supplementary Video 1), which works both as a stand-alone app and as an integrated resource in web repositories^2,59,60^. It offers a visual guide to navigate the steps required for the rigorous documentation of imaging experiments as sanctioned by 4DN-BINA-OME^11–13,59^.

### Micro-Meta App: intuitive microscopy experiment documentation

**Extended Data Fig. 2:**
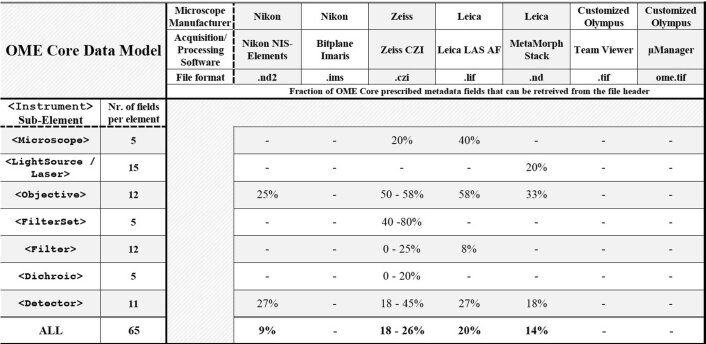
Table I | Percentage of OME Data Model Core <Instrument> element attributes that can be retrieved from image file headers and interpreted by BioFormats. The fraction of attributes associated with <Instrument> sub-elements (for example, <Microscope>, <Objective>, <Detector>, etc.,) of the core of the OME Data Model, whose values could be retrieved in the header of the raw image data files associated with the case studies presented in Fig. 2, Extended Data Figs. 6–9 and Supplementary Figs. 3–15, was determined and is reported above.

**Extended Data Fig. 3:**
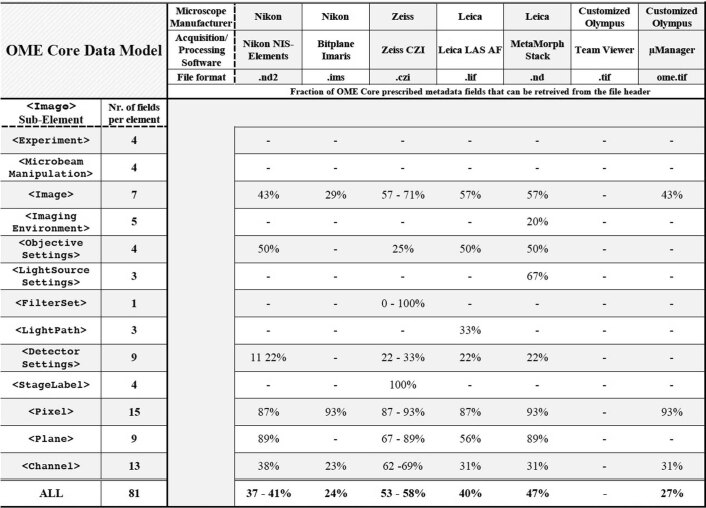
Table II | Percentage of OME Data Model Core <Image> element attributes that can be retrieved from image file headers and interpreted by BioFormats. The fraction of attributes associated with <Image> sub-elements (for example, <LightSourceSettings>, <Objective>, <Detector>, etc.,) of the core of the OME Data Model, whose values could be retrieved in the header of the raw image data files associated with the case studies presented in Fig. 2, Extended Data Figs. 6–9 and Supplementary Figs. 3–15, was determined and is reported above.

**Extended Data Fig. 4:**
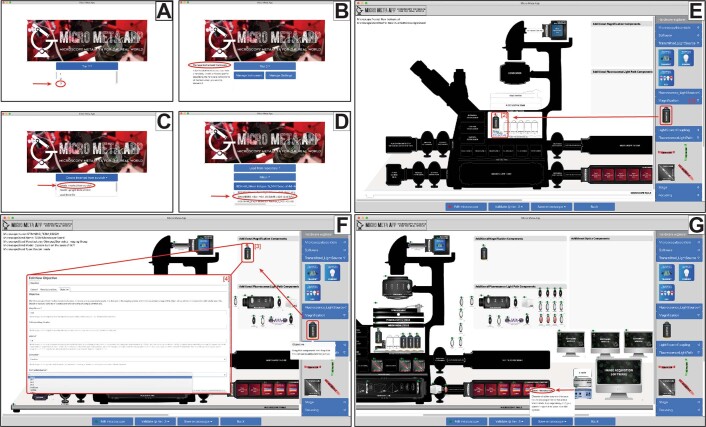
The Micro-Meta App is designed to aid in the collection of 4DN-BINA-OME sanctioned Microscope Hardware Specifications metadata. Micro-Meta App can be used as a stand-alone desktop application. Alternatively, it can be launched from a third-party portal such as the 4DN-Data Portal (Extended Data Fig. 10), where Microscope files can be stored as image-dataset attachments. In either situation: Α) After selecting the desired location for saving *Microscope.JSON* files for later use (not shown), before creating a new microscope file it is first necessary to select the desired Tier for the experiment at hand. Β) The *Manage Instrument* workflow in Micro-Meta App. The App can be used to create a structured *Microscope.JSON* file containing a description of the hardware components of a given microscopy Instrument based on the 4DN-BINA-OME ontology *<Instrument>* class model. C) A new microscope can be created totally from scratch by selecting either an Inverted or an Upright Microscope Stand. D) Alternatively, a previously saved *Microscope.JSON* file can be selected among those saved on an available repository and opened for further editing, such as in the depicted example. E) In this example, the *“Magnification”* drop- down menu is opened [1] and an *“Objective”* is dragged and snapped-in-place in the designated position [2] on the workspace. F) In this example, an additional “*Objective*” [3] is dragged onto the workspace and relevant metadata fields are entered using the designated metadata entry form [4]. G) Once all changes are entered, the microscope file can be saved to file or to the desired repository.

**Extended Data Fig. 5:**
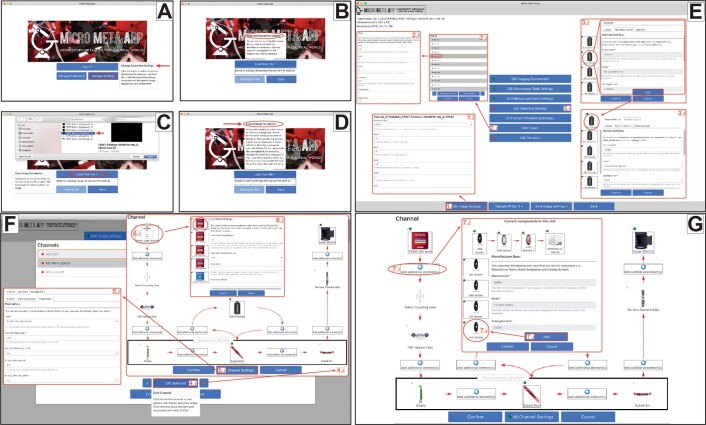
The Micro-Meta App utilizes the information contained in a previously generated Microscope metadata file and interactively aids in the collection of 4DN- BINA-OME sanctioned Image Acquisition settings metadata. Once a *Microscope.JSON* file is available (Figs. 1–2 and Extended Data Fig. 4), it is used as the basis for collecting the Image Acquisition Settings utilized for a specific microscopy experiment. After clicking on *Manage Settings* (A), the user first selects the Microscope metadata file describing the instrument used to acquire the image of interest (B), then the image file (C), and finally, if available, an existing Settings metadata file (D). From these files Micro-Meta App imports all relevant metadata for the user to inspect, edit and when missing enter from scratch. E) In the main window of *Manage Settings* the user accesses different sections of Settings metadata by launching the corresponding metadata collection windows. For example, the *Edit image settings* button [1.1] opens the metadata entry form [1.2] allowing to inspect and edit general *Image metadata*. In addition, the Edit Planes button [2.1] opens an interface where a given image Plane can be selected [2.2], and *Plane metadata* (for example, TimeStamp, ExposureTime) can be inspected and edited [2.3]. Finally, the *Edit Objective Settings* button [3.1] allows users to select the Objective that was used to acquire the image of interest, among those available in the Microscope file [3.2] and enter the relevant *Objective Settings* [3.3]. The same procedure is also used for the *Imaging Environment*, *Microscope Table*, *Microscope Stand*, and *Sample Positioning Settings* (not shown). F) After clicking the *Edit Channels* button in the main *Manage Settings* window (E), the list of *Channels* that were found in the image file header is displayed (top left) to be individually selected [4.1]. In the Channel interface [4.2] the *Edit Channel Settings* button [5.1] launches a general *Channel metadata* editing form [5.2], while an interactive GUI manages the different components of the channel’s *Lightpath*. In this example, the user clicks on *LightSource* [6.1] to select one of the available Light Sources present in the Microscope file, add it to the *Lightpath* and enter the associated settings that were applied during image acquisition [6.2]. G) Advanced features of the *Lightpath* are managed via one of the seven *Add additional element(s)* [7.1] buttons found at key locations along the lightpath. In the example, the *Add* [7.3] button is used to append an additional *Shutter* [7.4] after the existing *Shutter*, *Mirror, Dichroic* and *BeamSplitter* [7.2].

In the absence of tools that expedite image data documentation and QC, the metadata provided by manufacturers often does not align with existing minimal information criteria (that is, OME and BioFormats)^50,52^ and, as a consequence, is extremely variable, interfering with quality assessment, reproducibility and meaningful reuse of third-party datasets (Extended Data Figs. 2 and 3). Micro-Meta App, developed to address these unmet needs, consists of an interactive open-source and interoperable software tool to facilitate and (where possible) automate the annotation of light microscopy datasets. It provides a visual approach for documenting imaging experiments based on available OME-compatible community-sanctioned tiered systems (details in Supplementary Information) of specifications, such as 4DN-BINA-OME^11–13,53^. Thus, Micro-Meta App can adapt to varying levels of imaging complexity and to evolving data-models emerging from the community. To this aim, Micro-Meta App uses two parallel dataflows (Fig. 1):

1. In ‘Manage Instrument’ (hardware specifications; Figs. 1b and 2a and Extended Data Fig. 4) microscope users and custodians create accurate graphical depictions of microscope configurations by dragging-and-dropping icons representing hardware components onto the workspace while collecting relevant information scaling with microscope modality, experimental design, instrument-complexity and image analysis needs according to the 4DN-BINA-OME tier-level system^11–13,53^. From this, Micro-Meta App automatically generates interoperable Microscope.JSON files containing structured descriptions of the microscope Hardware Specifications (examples illustrated in Supplementary Information)^61^ that can be saved locally, and shared with other scientists via community repositories such as the 4DN-Data-Portal^2,29,60,62^, thus substantially lowering the burden of rigorous record-keeping and facilitating dissemination. Furthermore, such files can be imported in MethodsJ2 to automatically generate the Methods and Acknowledgement sections of scientific publications^14,15^.
2. To document the conditions used to produce specific image-datasets, ‘Manage Settings’ (Figs. 1c and 2b and Extended Data Fig. 5) (1) automatically extracts Hardware Specifications metadata from available Microscope.JSON files, (2) uses BioFormats^52^ to import OME metadata stored in the header of image files (Extended Data Figs. 2 and 3) and (3) interactively guides the user to enter missing, instrument-specific, tier-appropriate, 4DN-BINA-OME sanctioned ‘Settings’ metadata used during the relevant ‘Image Acquisition’ session. From this, the App generates interoperable Settings.JSON files containing comprehensive documentation of the ‘Image Acquisition Settings’ relative to individual microscopy datasets (examples illustrated in Supplementary Information)^61^. These files can be stored as described in (1) and associated with related Microscope.JSON files and image-datasets to ensure proper imaging experiment documentation.

Detailed descriptions of the functionality, implementation and documentation material of Micro-Meta App are available in Supplementary Information, Methods and ref. ^63^.

### Case studies: use at core facilities

**Extended Data Fig. 6:**
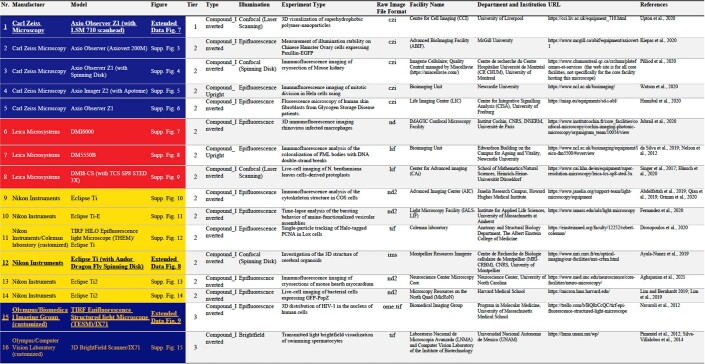
Table III | List of Microscopes documented using Micro-Meta App. List of key information regarding the microscopes, experiment types and image data file formats that were utilized to assess the feasibility of the Micro- Meta App approach to document microscopy experiments.

**Extended Data Fig. 7:**
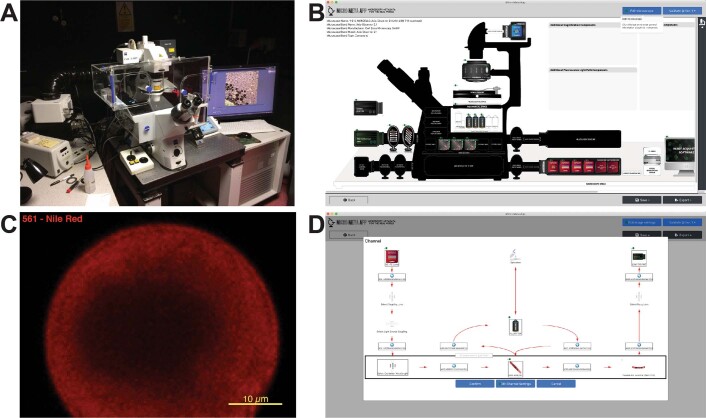
Micro-Meta App was utilized at the Centre for Cell Imaging of the University of Liverpool to document a Zeiss Axio Observer Z1 inverted microscope equipped with a LSM 710 confocal scan head and the settings that were applied to the microscope to acquire specific example image-datasets. Illustrated here is the use of Micro-Meta App for Tier 1 *(Minimum Information/Materials & Methods)*, 4DN-BINA- OME-specified^11–13^ documentation of: (A-B) *the Zeiss Axio Observer Z1 inverted microscope equipped with a LSM 710 confocal scan head*, owned by the Centre for Cell Imaging (CCI) of the University of Liverpool (Extended Data Fig. 6-Table III); and (C-D) the settings that were applied to the microscope for the acquisition of example published image-datasets^73^. A) Picture of the indicated microscope. B) Micro-Meta App-generated schematic representation of the indicated microscope. C) Particles composed of superhydrophobic polymer–nanoparticle composite (SPNC) material were analyzed by confocal microscopy to shed light on the differences in composite architecture between different coating polymers. Preparations of SiO2-polydimethylsiloxane (PDMS) coated SPNC (40–75 μm) particles were labelled by staining PDMS with Nile Red. Confocal 3D Z-stacks were acquired using the microscope indicated in Panels A and B. Depicted here is a slice from the middle (24 μm) of a Z-stack depicting a representative particle^73^. D) Micro-Meta App-generated schematic representation of the lightpath associated with the *561 Channel* utilized for the acquisition of the image in Panel C. To demonstrate the functionality and usability of the App and to assess the feasibility of the overall microscopy documentation approach, Micro-Meta App was tested independently at 16 sites (Extended Data Figs. 6–9 and Supplementary Figs. 3–15).

**Extended Data Fig. 8:**
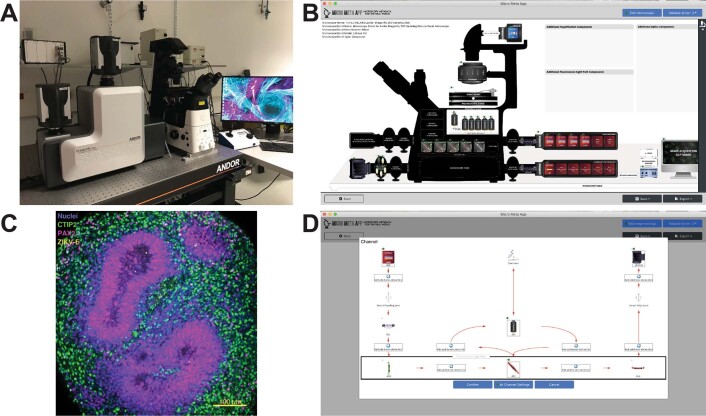
Micro-Meta App was utilized at the Montpellier Resources Imagerie of the University of Montpellier to document a Andor Dragonfly Spinning Disk confocal microscope system and the settings that were applied to the microscope to acquire specific example image-datasets. Illustrated here is the use of Micro-Meta App for Tier 2 *(Advanced Quantification and/or Live cell Imaging)*, 4DN-BINA-OME- specified^11–13^ documentation of: (A-B) the *Andor Dragonfly Spinning Disk confocal microscope system (with Nikon Eclipse Ti inverted compound microscope stand)* owned by the Montpellier Resources Imagerie at the Centre de Recherche de Biologie cellulaire de Montpellier (MRI-CRBM) of the University of Montpellier (Extended Data Fig. 6-Table III); and (C-D) the settings that were applied to the microscope for the acquisition of example published image-datasets^70^. A) Picture of the indicated microscope. B) Micro-Meta App-generated schematic representation of the indicated microscope. C) Cerebral organoids were cocultured with ZIKV-infected monocytes, fixed, permeabilized, stained with DAPI to detect all cellular nuclei (405 channel, visualized in blue) anti-CTIP2 (488 channel, visualized in green), anti-PAX6 (568 channel, visualized in magenta) and anti- ZIKV2-E (Flavivirus group antigen Antibody (D1-4G2-4-15 (4G2); 640 channel, visualized in yellow), and clarified. 3D Z- stacks of representative organoids were acquired using the indicated microscope (Panels A and B) equipped with a 20x, NA 0.8, air Nikon objective. The displayed image corresponds to a representative Z-plane^70^. D) Micro-Meta App-generated schematic representation of the lightpath associated with the *488 Channel* utilized for the acquisition of the image in Panel C. To demonstrate the functionality and usability of the App and to assess the feasibility of the overall microscopy documentation approach, Micro-Meta App was tested independently at 16 sites (Extended Data Figs. 6–9 and Supplementary Figs. 3–15).

**Extended Data Fig. 9:**
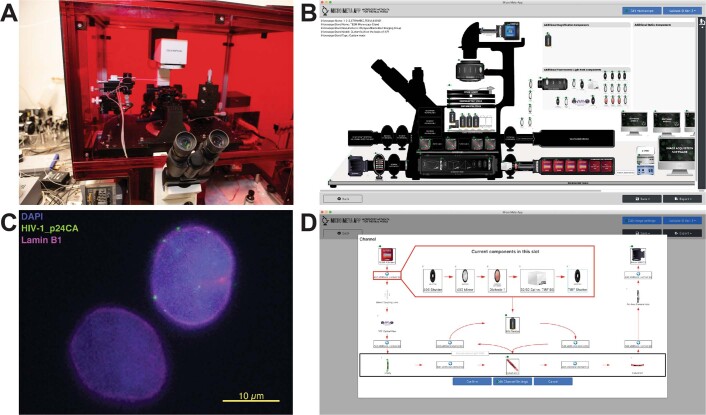
Micro-Meta App was utilized at UMass Medical School to document a custom built TIRF Epifluorescence Structured light Microscope and the settings that were applied to the microscope to acquire specific example image-datasets. Illustrated here is the use of Micro-Meta App for Tier 3 *(Manufacturing/Technical Development/Full Documentation)*, 4DN-BINA-OME-specified^11–13^ documentation of: (A-B) the *custom build TIRF Epifluorescence Structured light Microscope* (TESM; based on Olympus IX71) developed, built and owned by the Biomedical Imaging Group^79^ at the Program in Molecular Medicine of the UMass Medical School (Extended Data Fig. 6-Table III); and (C-D) the settings that were applied to the microscope for the acquisition of example images. A) Picture of the indicated microscope. B) Micro-Meta App- generated schematic representation of the indicated microscope. C) TZM-bl human cells were infected with HIV-1 retroviral vectors, fixed and permeabilized before staining with mouse-anti-p24 primary followed by DyLight488-anti-Mouse secondary antibodies, to detect HIV-1 viral Capsid. In addition, cells were counterstained using rabbit-anti-LaminB1 primary followed by DyLight649-anti-Rabbit secondary antibodies, to visualize the nuclear envelope and with DAPI to visualize the nuclear chromosomal DNA. Displayed is a representative image obtained using the indicated microscope (Panels A and B)^79^. D) Micro-Meta App-generated schematic representation of the lightpath associated with the *DAPI channel* utilized for the acquisition of the image in Panel C. E) The insert shows a detail of the optical path followed by the 405-laser beam, in which the TIRF lightpath was used for epifluorescence illumination by orienting the beam perpendicular to the specimen plane. Specifically, in this portion of the lightpath the 405-laser beam passes through an electronically controlled shutter (*405 Shutter*), a mirror (*405 Mirror*), a dichroic filter (*Dichroic 1*), a 50/50 beam splitter *(50/50 Epi* vs. *TIRF BS*) and finally a shutter to control the TIRF lightpath illumination (*TIRF Shutter*). Example Microscope.JSON, Settings.JSON and associated image data file for this use case are available at: https://doi.org/10.5281/zenodo.4891883. To demonstrate the functionality and usability of the App and to assess the feasibility of the overall microscopy documentation approach, Micro-Meta App was tested independently at 16 sites (Extended Data Figs. 6–9 and Supplementary Figs. 3–15).

To demonstrate feasibility and test usability, Micro-Meta App was employed, with minimal initial training, at 16 partnering core facilities (Extended Data Fig. 6) to document both example microscope instrumentation and the settings used for the acquisition of exemplar image-datasets (Fig. 2, Extended Data Figs. 7–9 and Supplementary Figs. 3–15)^64–83^. The most striking result (detailed in Supplementary Information) was that, in comparison with the baseline represented by BioFormats alone (Extended Data Figs. 2 and 3), the use of Micro-Meta App considerably increased the uniformity of reported metadata fields, facilitating comparison of image data within and across different microscopes and imaging experiments. In addition, since the App’s data model is defined dynamically on the basis of shared and evolving community specifications, the use of this method maximizes reproducibility, quality and value, while minimizing effort on the part of individual scientists. Example Microscope.JSON, Settings.JSON and image files produced for the use case in Extended Data Fig. 9 are publicly available on Zenodo as illustrated in the Data Availability section and in Supplementary Information^61^.

### Case studies: integration to 4DN-Data-Portal

**Extended Data Fig. 10:**
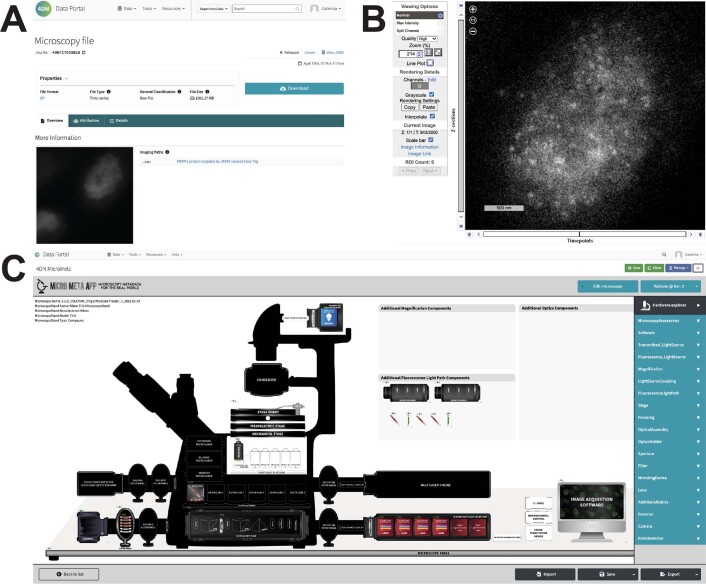
Micro-Meta App is integrated in the 4DN-Data Portal for the documentation of imaging experiments. In order to facilitate the full documentation of microscopy experiments (A), ensure the quality, reproducibility and value of image data (B), and facilitate their integration with genomic data, Micro-Meta App (C) was incorporated in the 4DN-Data Portal and made an integral part of the imaging experiment documentation and quality control workflow developed by the 4DN-Data Coordination and Integration Center (DCIC) of the NIH-funded 4DN Consortium^2,59,60,62^. For this use the App’s data flow was adapted to allow the direct ingestion of the content of the JSON Microscope- and Settings-files into the portal database, which allows individual fields to be utilized for filtering and searching purposes and to be visualized directly on the portal. Data for this figure is available publicly on the 4DN-Data Portal^59,60^ as follows: A) https://data.4dnucleome.org/files-microscopy/4DNFI7639BEB/; B) https://omero.hms.harvard.edu/pathviewer/vanilla-viewer/975042/; C) https://data.4dnucleome.org/microscope-configurations/28f1c0f2-d903-4761-93c6-dd3994db3462/.

An initial impetus for the development of Micro-Meta App was the need to expedite and, where possible, automate the rigorous reporting of imaging experiments and QC procedures for the purpose of integrating 4DN imaging and omics experiments^2^. Thus, the Micro-Meta App was embedded into the 4DN-Data-Portal (Extended Data Fig. 10)^59,60,62^ and the content of the Microscope .JSON file was integrated directly into the portal’s database. This allows microscopy metadata associated with individual experiments to be used for searching, filtering and visualization purposes.

### Case studies: teaching with Micro-Meta App

Micro-Meta App provides a digital representation of freely configurable microscopes, ideal for microscopy custodians to provide users with a detailed inventory of all available microscopes and for teaching purposes (Supplementary Video 1). Micro-Meta App was used by graduate students at UMass Medical School^84^ for working on (1) specific problem sets and (2) self-driven exploration of microscope components, functions and imaging modalities. The success of these pilots indicates that Micro-Meta App could be used in online teaching-modules (for example, GlobalBioImaging (GBI) Training Resources)^85^ for familiarizing users with the intricacies of specific instrument hardware configurations and for the interactive application of microscopy concepts.

### Future directions

Micro-Meta App has been developed and will continue to evolve in close collaboration with communities that include 4DN^2,3^, BINA^5^, GBI^86^ and QUAREP-LiMi^6–8,87^. Ongoing efforts include:

1. Outreach and education: to increase awareness and promote adoption of microscopy documentation and QC, and disseminate the use of Micro-Meta App, we have initiated an extensive outreach effort directed towards microscope users, custodians and manufacturers, which includes university classes (Case Studies), online training and workshops^88–90^. This will be augmented with specialized inperson courses as circumstances allow and in close collaboration with our community partners^91,92^.
2. Creation of Instrument and Hardware components databases: while engaging with microscopy manufacturers to ensure the full automation of light microscopy data provenance and QC reporting, it will be necessary to engage the community to reduce the burden imposed on individual microscope custodians and users that need to document similar imaging experiments, therefore maximizing their adoption of community standards. For this purpose, and echoing 4DN^59,60,62^, we are developing exchange-sites for microscopy metadata JSON files, similar to ImageJ/Fiji plugin repositories. Furthermore, integration with the Research Resource ID (RRID) effort^93^ could promote the recognition of microscope configurations as a quantifiable scientific output, providing credit to the work of imaging scientists.
3. Further integration with MethodsJ2: Micro-Meta App will be extended to automatically generate text for scientific publications and MethodsJ2 (refs. ^14,15^) will be adapted to use Micro-Meta App Settings.JSON files as sources of image acquisition settings and QC metadata.
4. Further OMERO and OMERO.mde integration: a pilot Micro-Meta App OMERO plugin is available^94^. Future development will include extracting experimental metadata developed using OMERO.mde^16,17^ and saving 4DN-BINA-OME metadata^11–13^ as collections of key-value pairs associated with individual OMERO image-datasets.
5. Implementation of additional microscopy modalities and QC: currently, the App implements the Core OME data model and the 4DN-BINA-OME Basic extension^11–13^. Efforts to implement the Confocal and Advanced as well as the Calibration and Performance 4DN-BINA-OME extensions are underway. As a proof-of-concept, we are collaborating with QUAREP-LiMi^95^ to automatically annotate imaging datasets with calibration metrics calculated using the open-hardware Meta-Max^96^ calibration tool.

## Conclusions

Easily accessible and facile tools such as Micro-Meta App, MethodsJ2 and OMERO.mde are essential for microscope custodians and users to see image data documentation and QC as routine tasks in their imaging workflow, therefore promoting better quality, reproducibility and value for imaging data. In addition, reaching this goal will entail partnering with manufacturers to promote the automated interpretation of metadata stored in image file headers, the development of community-wide repositories for microscopy hardware metadata specifications and the automated annotation of datasets to be uploaded in imaging data repositories^30^. Thus, key support from funding agencies and institutions will ultimately lead to automating all aspects of the process used by members of the community to annotate and upload metadata-rich imaging datasets to both local and public repositories^38,40,51^. As an added advantage, full documentation of the provenance and QC of imaging experiments will be key for the development of pipelines to integrate images and their metadata with -omics data from the same experiment, such as is underway as part of 4DN.

## Methods

### Software implementation

Micro-Meta App is available in two JavaScript (JS) implementations. The first was designed to facilitate integration of the software into third-party web portals, such as the 4DN-Data Portal)^2,59,60,62,97^ and OMERO^94^, and was developed using the JS React library, which is used widely to build web-based user interfaces. Starting from this version, a stand-alone version of the App was developed by wrapping the React implementation using the JS Electron library, with the specific purpose of lowering the barrier of adoption of the tool by laboratories that do not have access to, or prefer not to use, imaging databases.

#### Dependencies

For the Micro-Meta App to work, the following elements are generated in advance as described in the following sections and made available via GitHub:

1. JSON schema: a JSON file or a series of files that define the underlying schema, which are used to construct the graphical user interface (GUI) of the application on the basis of the 4DN-BINA-OME Microscopy Metadata specifications^11–13^.
2. Dimensions and coordinates: a JSON file that codifies the dimensions of the canvases used by the Manage Instrument GUI of the Micro-Meta App as well as the position on the canvas occupied by icons representing each microscope hardware component.
3. Icons: a series of SVG files, each containing an icon representing the individual hardware components.

In addition, the stand-alone version of Micro-Meta App depends on the 4DN Microscopy Metadata Reader^98^, which implements the BioFormats library^52^ to allow the user to import known OME metadata directly from the file header of an image data file of interest. To maximize flexibility, these elements can be customized to meet the needs of individual users.

##### XSD to JSON schema converter

The main function of this Java-encoded component^99^ is to transform the XML schema definition (XSD) implementation of the 4DN-BINA-OME data model^11–13,100^ into a JSON-based schema, which is subsequently ingested by Micro-Meta App to automatically generate the software GUI and the associated data insertion forms. The XSD to JSON schema converter middleware uses the Xerces2 Java XML Parser^101^ and W3C Java XML bindings libraries^102^ to navigate the XSD schema, and produces two kinds of version-aware JSON files:

1. A comprehensive JSON file containing an array of schemas for all necessary individual components that constitute the 4DN-BINA-OME data model (for example, Objective, Filter or Detector). This comprehensive JSON file is made available on GitHub and is designed specifically to facilitate the remote loading of the schema by web portal embedded React implementations of the Micro-Meta App. This schema is available as an individual file on GitHub (https://github.com/WU-BIMAC/4DNMetadataSchemaXSD2JSONConverter/blob/master/latest/fullSchema.json).
2. A series of JSON files, each containing the schema of individual components, which were designed to be employed by the Electron implementation of the App. These individual schema files are available within a subdirectory of the main repository on GitHub (https://github.com/WU-BIMAC/4DNMetadataSchemaXSD2JSONConverter/tree/master/latest/schemas).

The middleware was designed specifically to maximize flexibility and extensibility. As such, the software allows the introduction of implementation-specific modifications of the resulting JSON schema so that it can be adapted for special purposes. For example, the introduction of a ‘Version’ field allows validation of whether the data being saved is compatible with the specific version of the schema being employed. As a further example, the introduction of the ‘Category’ field allows the organization of different components in specific submenus across the sidebar. To facilitate the evolution of the model while ensuring back-compatibility, the GitHub repository supports versioning by storing all revisions of the output JSON schema.

##### TXT to JSON dimensions converter

This Java-encoded component^99^ is used to process an input text file containing the dimensions of the Manage Instrument canvas of Micro-Meta App alongside the desired *x*,*y* positions where each icon has to be placed. As a result, the software produces a Dimensions and Coordinate JSON file that is made available for remote loading from GitHub (https://github.com/WU-BIMAC/4DNMetadataSchemaXSD2JSONConverter/tree/master/latest/dimensions)^99^ and is ready to be used to implement the ‘snap-in-place’ functionality of the software.

##### ICON

Scalable vector graphic (SVG) files containing icons representing the different microscope hardware components were generated specifically for this project and are made available in a version-aware manner for remote loading from GitHub (https://github.com/WU-BIMAC/4DNMetadataSchemaXSD2JSONConverter/tree/master/latest/images)^99^. Custom-made icons can be similarly generated by developer users and manufacturers for representation of their hardware components within the application.

##### Microscopy metadata reader

This software is written in JAVA to fully take advantage of the BioFormats library and the OME-XML metadata structure^98^. Using these two dependencies, this software accesses all the OME-compatible metadata present in user-selected images and maps it to the 4DN-BINA-OME microscopy metadata specifications^11–13^ that extend the OME Data Model^50,52^ to produce a temporary, Micro-Meta App-compatible JSON object that can be read by Micro-Meta App. The object is then passed on to the Micro-Meta App and read by the Manage Settings section of the App to prepopulate the corresponding metadata fields.

#### JS React implementation of Micro-Meta App

This is the core implementation of the Micro-Meta App, and it is the starting point for embedding it into a third-party web portal, and for wrapping it into Electron for local execution^97^. This component ingests JSON schema files, JSON Dimension and Coordinate files and Icon images produced as described above and uses a series of custom React classes to produce a set of individual windows composing the GUI. Specifically, these windows can be categorized into two main sections: the Manage Instrument and the Manage Settings (Figs. 1 and 2 and Supplementary Figs. 2 and 3). In the Manage Instrument section, the application employs a Canvas setup that provides the flexibility to incorporate as many icon elements as necessary to describe the hardware components of any given microscope. For this purpose, the toolbar is generated dynamically as dictated by the underlying JSON schema produced as described above and by the selected tier level. On this basis, elements present in each of the graphical menus present on the sidebar can be dragged to the canvas and dropped either to a custom position specified by the user, or they can be snapped-in-place as defined by the position and dimension file produced by the Dimension Converter component.

In the Manage Settings, the application builds a series of nested windows that are launched by individual buttons allowing the user to select individual hardware components and enter specific settings for each of them. Of particular interest is the Channel interface in which the user can define the LightPath configuration associated with each channel of a given image by following a predefined graphical flowchart (Supplementary Fig. 3d).

#### JS Electron implementation of Micro-Meta App

This implementation of the Micro-Meta App is encoded in JS^103^. It uses the Electron library to wrap the React implementation of Micro-Meta App with all necessary schemas, icon images and position/dimension to ensure its correct functionality. This discrete executable file can interact directly with the underlying operating system and launch the software from the local file system.

### Beta testing

Micro-Meta App was developed in the context of community efforts organized around the 4DN Consortium need for imaging data dissemination and integration with omics datasets^2,3^ and the BINA^5^ effort to improve rigor, QC and reproducibility in light microscopy. As part of this effort, several core facilities were identified to serve as reference beta-testing sites for the Micro-Meta App (a subset of the facilities listed in Extended Data Fig. 6). To this aim, the stand-alone JS Electron implementation of Micro-Meta App was deployed locally and microscope custodians at individual beta-testing sites were trained both on the use of the App and on bug and feature request reporting. Such feedback was collected either directly or by taking advantage of the GitHub issue-reporting feature and incorporated into the main development branch in a close-iterative cycle ahead of the release of the initial production version of the software

### Reporting Summary

Further information on research design is available in the Nature Research Reporting Summary linked to this article.

## Online content

Any methods, additional references, Nature Research reporting summaries, source data, extended data, supplementary information, acknowledgements, peer review information; details of author contributions and competing interests; and statements of data and code availability are available at 10.1038/s41592-021-01315-z.

## Supplementary information

Supplementary Information Supplementary 4DN-BINA-OME tier-system description, Micro-Meta App description (including detailed descriptions of the ‘Manage Instrument’, Image Acquisition ‘Manage Settings’ sections of the App), case studies (including detailed descriptions of the ‘use at core facilities’ and ‘teaching with Micro-Meta App’ case studies), video 1, Figs. 1–15 and references.

Reporting Summary

Supplementary Video Video 1 was used as an introduction to Micro-Meta App for use by Graduate Students at the University of Massachusetts Medical School^84^ is also available at: https://vimeo.com/557097919. Images labeled with (*) and (**) at minutes 0:16–0:33 of Video 1 are used with permission from the author from Figs. 5 and 6 of Smith et al.^100^.

## Data Availability

Data associated with this manuscript is available as follows: (1) Example Microscopy Metadata JSON files and associated image data file related to the use case presented in Fig. 2 and Extended Data Fig. 9 are publicly available on Zenodo at: 10.5281/zenodo.4891883. These files were produced using Micro-Meta App at UMass Medical School to document the acquisition of the FSWT-6hVirus-10minFIX-stk_4-EPI.tif.ome.tif example image file using the TIRF Epifluorescence Structured Illumination Microscope (TESM)^79^ custom built by the Biomedical Imaging Group. These files demonstrate the usability of Micro-Meta App to document microscopy experiments. (2) Example datasets associated with Extended Data Figs. 7–8 and Supplementary Figs. 3–15 and used at 16 different imaging core facilities to evaluate the functionality and test the usability of Micro-Meta App, can be made available upon request from the corresponding author. These exemplar image data files were not produced to test hypotheses or reach conclusions that are part of this study. Rather, they were used successfully as case studies to test the feasibility of the Micro-Meta App approach. (3) Data associated with Extended Data Fig. 10 are available publicly on the 4DN-Data Portal as follows: panel 10ahttps://data.4dnucleome.org/files-microscopy/4DNFI7639BEB/; 10bhttps://omero.hms.harvard.edu/pathviewer/vanilla-viewer/975042/; 10chttps://data.4dnucleome.org/microscope-configurations/28f1c0f2-d903-4761-93c6-dd3994db3462/. (4) Supplementary Video 1 is also available publicly at: https://vimeo.com/manage/videos/604291798

## Code availability

This manuscript describes the Micro-Meta App software tool whose function is to facilitate the collection of Microscopy Metadata that can be used to document the hardware specifications, image acquisition settings and QC metrics used to produce high-quality, reproducible and valuable light microscopy image data. Micro-Meta App is available in two JS implementations that are described in detail above. To promote the adoption and reuse of Micro-Meta App, the executables and source code for both JS-React and -Electron implementations of Micro-Meta App are available on GitHub^97,103^. In addition, a website describing Micro-Meta App^104^ was developed alongside complete documentation and tutorials^105^. More specifically, as listed in the Reporting Summary document associated with this publication, and on the README file on GitHub (https://github.com/WU-BIMAC/MicroMetaApp-Electron), the software is available as follows: (1) *Project name*: Micro-Meta App. (2) *Project home page*: https://github.com/WU-BIMAC/MicroMetaApp.github.io. (3) *Documentation* (including video tutorials): https://micrometaapp-docs.readthedocs.io/en/latest/index.html. *Note*: if you intend to use the Micro-Meta App on MacOS you might encounter difficulties unzipping and launching the MacOS Zip. To address these issues please follow the special instructions specified in this VIDEO: https://vimeo.com/529609242. (4) *Executable*: https://github.com/WU-BIMAC/MicroMetaApp-Electron/releases/latest (10.5281/zenodo.4750765). (5) *Source code*: (i) https://github.com/WU-BIMAC/MicroMetaApp-Electron (10.5281/zenodo.4750765); (ii) https://github.com/WU-BIMAC/MicroMetaApp-React (10.5281/zenodo.4889259). (6) *Operating system(s)*: (i) Windows ×32; (ii) Windows ×64 (tested on Win 7 Pro v.6.1.760; Win 8.1 v.6.3.9600; Win 10 Home v.10.0.19040–10.0.19041–10.0.19042; Win 10 Pro v.10.0.19041–10.0.19042; Win 10 Enterprise v.10.0.18362, 10.0.19041 and 10.0.19042); (iii) MacOS (tested on High Sierra v.10.13.6; Mojave v.10.14.6; Catalina v.10.15.7; Big Sur v.11.0–11.1–11.2–11.3–11.3.1–11.4–11.5 Beta). (7) *P**rogramming* *language*: JS and Java. (8) *Other requirements*: Java v.1.8.0. In addition, see dependencies listed in Methods and the Supplementary Information. (9) *License*: GNU GPL v.3 (https://www.gnu.org/licenses/gpl-3.0.html).
