# Author Correction: Micro-Meta App: an interactive tool for collecting microscopy metadata based on community specifications

**DOI:** 10.1038/s41592-021-01388-w

**Published:** 2021-12-24

**Authors:** Alessandro Rigano, Shannon Ehmsen, Serkan Utku Öztürk, Joel Ryan, Alexander Balashov, Mathias Hammer, Koray Kirli, Ulrike Boehm, Claire M. Brown, Karl Bellve, James J. Chambers, Andrea Cosolo, Robert A. Coleman, Orestis Faklaris, Kevin E. Fogarty, Thomas Guilbert, Anna B. Hamacher, Michelle S. Itano, Daniel P. Keeley, Susanne Kunis, Judith Lacoste, Alex Laude, Willa Y. Ma, Marco Marcello, Paula Montero-Llopis, Glyn Nelson, Roland Nitschke, Jaime A. Pimentel, Stefanie Weidtkamp-Peters, Peter J. Park, Burak H. Alver, David Grunwald, Caterina Strambio-De-Castillia

**Affiliations:** 1 Program in Molecular Medicine, UMass Chan Medical School, Worcester, MA USA; 2 Department of Biomedical Informatics, Harvard Medical School, Boston, MA USA; 3 Advanced BioImaging Facility (ABIF), McGill University, Montreal, Quebec Canada; 4 RNA Therapeutics Institute, UMass Chan Medical School, Worcester, MA USA; 5 Janelia Research Campus, Howard Hughes Medical Institute, Ashburn, VA USA; 6 Institute for Applied Life Sciences, University of Massachusetts, Amherst, MA USA; 7 Department of Anatomy and Structural Biology, Gruss-Lipper Biophotonics Center, Albert Einstein College of Medicine, Bronx, NY USA; 8 BioCampus Montpellier (BCM), University of Montpellier, CNRS, INSERM, Montpellier, France; 9 Institut Cochin, Inserm U1016-CNRS UMR8104-Université de Paris, Paris, France; 10 Center for Advanced Imaging, Heinrich-Heine University Duesseldorf, Düsseldorf, Germany; 11 UNC Neuroscience Microscopy Core Facility, Department of Cell Biology and Physiology, Carolina Institute for Developmental Disabilities, and UNC Neuroscience Center, University of North Carolina, Chapel Hill, NC USA; 12 Department of Biology/Chemistry and Center for Cellular Nanoanalytics, University Osnabrück, Osnabrück, Germany; 13 MIA Cellavie Inc., Montreal, Quebec Canada; 14 Bioimaging Unit, Newcastle University, Newcastle upon Tyne, UK; 15 UNC Neuroscience Microscopy Core Facility, Carolina Institute for Developmental Disabilities, and UNC Neuroscience Center, University of North Carolina, Chapel Hill, NC USA; 16 Center for Cell Imaging, University of Liverpool, Liverpool, UK; 17 Microscopy Resources of the North Quad, University of Harvard Medical School, Boston, MA USA; 18 Life Imaging Center and Signalling Research Centres CIBSS and BIOSS, University of Freiburg, Freiburg, Germany; 19 Laboratorio Nacional de Microscopía Avanzada, Instituto de Biotecnología, Universidad Nacional Autónoma de México, Cuernavaca, Mexico

**Keywords:** Confocal microscopy, Data publication and archiving, Standards, Software, Wide-field fluorescence microscopy

Correction to: *Nature Methods* 10.1038/s41592-021-01315-z, published online 3 December 2021.

In the version of this article initially published, the affiliation for author Roland Nitschke was incorrectly formulated. The affiliation has been corrected to read “Life Imaging Center and Signalling Research Centres CIBSS and BIOSS, University of Freiburg, Freiburg, Germany” in the online version of the article.

