# Supplementary material for: Micro-Meta App: an interactive tool for collecting microscopy metadata based on community specifications
Source: Nat Methods. 2021 Dec 3;18(12):1489–95. doi: 10.1038/s41592-021-01315-z (PMC8648560; doi:10.1038/s41592-021-01315-z)
Supplement: Supplementary file 1 — Supplementary 4DN-BINA-OME tier-system description, Micro-Meta App description (including detailed descriptions of the ‘Manage Instrument’, Image Acquisition ‘Manage Settings’ sections of the App), case studies (including detailed descriptions of the ‘use at core facilities’ and ‘teaching with Micro-Meta App’ case studies), video 1, Figs. 1–15 and references. [file 41592_2021_1315_MOESM1_ESM.pdf]

nature portfolio

https://doi.org/10.1038/s41592-021-01315-z

Supplementary information

Micro-Meta App: an interactive tool for collecting microscopy metadata based on community specifications

In the format provided by the authors and unedited

Micro-Meta App: an interactive tool for collecting microscopy metadata based on community specifications

Alessandro Rigano[^*], Shannon Ehmsen[*], (https://orcid.org/0000-0001-8835-787X) Serkan Utku Öztürk[*], Joel Ryan, Alexander Balashov[*], Mathias Hammer[^*], (https://orcid.org/0000-0002-2289-0652) Koray Kirli[*], (https://orcid.org/0000-0001-7471-2244) Ulrike Boehm[#^], (https://orcid.org/0000-0003-1622-663X) Claire M. Brown[#^], Karl Bellve', (https://orcid.org/0000-0003-3883-8215) James J. Chambers[#^], (https://orcid.org/0000-0003-3417-0713) Andrea Cosolo[*], (https://orcid.org/0000-0002-7367-9603) Robert A. Coleman[*], (https://orcid.org/0000-0001-5965-5405) Orestis Faklaris[#^], (https://orcid.org/0000-0002-4651-3480) Kevin E. Fogarty, (https://orcid.org/0000-0001-5069-0730) Thomas Guilbert[^], (https://orcid.org/0000-0001-7374-8245) Anna B. Hamacher, (https://orcid.org/0000-0001-6853-1228) Michelle S. Itano[^], Daniel P. Keeley, (https://orcid.org/0000-0001-6523-7496) Susanne Kunis[^], (https://orcid.org/0000-0002-8783-8599) Judith Lacoste[#^], (https://orcid.org/0000-0002-3853-1187) Alex Laude[#^], Willa Y. Ma, (https://orcid.org/0000-0002-2392-8640) Marco Marcello[^], (https://orcid.org/0000-0002-5983-2296) Paula Montero-Llopis[^], (http://orcid.org/0000-0002-1895-4772) Glyn Nelson[#^], (https://orcid.org/0000-0002-9397-8475) Roland Nitschke[#^], (https://orcid.org/0000-0001-8569-0466) Jaime A. Pimentel[#^], (https://orcid.org/0000-0001-7734-3771) Stefanie Weidtkamp-Peters, (https://orcid.org/0000-0001-9378-960X) Peter J. Park[*], (https://orcid.org/0000-0002-5019-7652) Burak H. Alver[*], (https://orcid.org/0000-0001-9067-804X) David Grunwald[^*], (https://orcid.org/0000-0002-1069-1816) Caterina Strambio-De-Castillia[#^*&]

# Members of the Bioimaging North America Quality Control and Data Management Working Group (BINA-DM-QC-WG)
^ Member of Quality Assessment and Reproducibility for Light Microscopy (QUAREP-LiMi)
** Member of the 4D Nucleome Network*
& Corresponding Author (caterina.strambio@umassmed.edu)

SUPPLEMENTARY 4DN-BINA-OME TIER-SYSTEM DESCRIPTION

The 4DN-BINA-OME tier system[1–4] is used to decide what reporting guidelines are most appropriate for a given experiment-design, instrument-complexity and image analysis needs. A brief summary of the Tiers is provided here:

- Tier 1 is appropriate for reporting effects that require simple or no quantification, and represents the minimum information to be used for documenting imaging experiments and reporting in scientific publications.
- Tier 2 is appropriate for reporting quantitative effects that require advanced quantification such as localization of single molecules and tracking of intracellular dynamics
- Tier 3 is appropriate for the full documentation of microscope instrumentation, image acquisition and quality control and it is recommended for technology developers and instrument manufacturers.

SUPPLEMENTARY MICRO-META APP DESCRIPTION

Manage Instrument Hardware

As also described in detail in preprint[5], the *Manage Instrument* hardware section of Micro-Meta App (Figures 1B and 2A and Extended Data Figure 4) is composed of the following steps :

1) After launching the application, the user selects an appropriate Tier to be used (Extended Data Figure 4A) to document a given imaging experiment as determined by 4DN-BINA-OME tiered specifications[2–4] and launches the ***Manage Instrument*** modality of Micro-Meta App by clicking the appropriate button (Extended Data Figure 4B). Because Micro-Meta App was specifically designed to be tier-aware, Micro-Meta App automatically displays only metadata fields that are specified by 4DN-BINA-OME[2–4] to belong to the tier that was selected upon launching the App (Extended Data Figure 4A), thus massively reducing the documentation burden. In addition, to increase flexibility, the tier-level utilized for validation can be modified dynamically after opening the main Manage Instrument workspace. This way, the user can, for example, be presented with all Tier 2 appropriate fields while being required to only fill in Tier 1 fields for validation (see also point 3 ii).
2) Once entered in the ***Manage Instrument*** section, the user is given the option of selecting one of three different methods for managing an instrument (Extended Data Figure 4C and D):
   i) By selecting one of the two *Create from scratch* options (i.e., *Create Inverted from scratch* or *Create Upright from scratch*), the user is presented with a blank microscope canvas of the selected type to work with (Extended Data Figure 4E).
   ii) When selecting *Load from file*, the user is asked to select a pre-existing *Microscope.JSON* (example available on Zenodo as illustrated in the Data Availability section of Online Methods)[6] file from the local file system. Such *Microscope.JSON* files could, for example, be a template file that was created by microscope custodians at the local microscopy core facility, shared by a colleague, or downloaded from a repository for local use.
   iii) Finally, a pre-existing *Microscope.JSON* file that has already been processed and saved to the local Micro-Meta App's *Home Folder* can be loaded for further editing using the *Load from repository* modality. Here existing *Microscope.JSON* files are listed by Manufacturer and by Instrument Name to facilitate the selection of the appropriate file (Extended Data Figure 4D).
3) Regardless of the chosen ***Manage Instrument*** modality, in the next step, the user is presented with the main instrument management workspace where they can perform the following actions:
   i) In the top bar, the user can select a different tier-level for validation with respect to the one that had been selected upon entering the current Micro-Meta App session (Extended Data Figure 4E). This feature allows the user to fill additional fields while not being required to provide all mandatory fields for a given tier level.
   ii) By clicking the *Edit microscope* button (Extended Data Figure 4E), the user can then enter attributes that refer to the instrument in general and that allow the description of the Microscope Stand the instrument is built upon. Upon exiting the *Edit Microscope* GUI, the application signals validation by changing the color of the dot on the button from red (i.e., incomplete) to green (i.e., complete and validated).

iii) By clicking the sidebar *Hardware Navigation* selection menus, the user can identify a given hardware component and drag it to the appropriate position on the instrument canvas, or to one of the top right generic "drawers" that can accommodate free-floating components that do not have a pre-existing position on the canvas (i.e., additional Objectives that do not fit in the objective turret). In the example depicted, a blank Objective component is selected from the *Magnification* menu (Extended Data Figure 4E, [1]) and dragged to one of the objective-slots on the canvas, where it "snaps in place" upon release (Extended Data Figure 4E, [2]).

iv) Once a component has been placed on the canvas, the icon is highlighted with a red dot to signal that the attributes have not been yet filled in and validated. Thus, the user is alerted about which microscope components are in need of attention and can quickly identify which icon to work on. By clicking on the icon (Extended Data Figure 4F, [3]), the user gains access to different tabs, each containing simple forms that display the required, tier-appropriate, metadata fields sanctioned by 4DN-BINA-OME (Extended Data Figure 4F, [4])[2–4] . To further increase usability, the *Confirm* button found at the bottom of each window can be used to automatically jump to mandatory fields that have to be filled before exiting. Upon completing all required fields, the icon is highlighted with a green dot making it easier for the user to assess documentation progress.

4) Once all appropriate microscope hardware components for a given instrument have been added to the canvas and appropriately attended to, the resulting tier-specific microscope *Hardware Specification* descriptions are then output as structured and interoperable *Microscope.JSON* files (example available on Zenodo as illustrated in the Data Availability section of Online Methods)[6]. These files can be Saved to the local Micro-Meta App working folder (Extended Data Figure 4G) or used by existing third-party databases, such as the 4DN Data Portal [7,8] (Extended Data Figure 10), for later utilization during the ***Manage Setting***s modality of the Micro-Meta App (see next section). In addition, such files can be imported in MethodsJ2 and used to automatically generate the Methods and Acknowledgement sections of scientific publications as described in a parallel manuscript[9,10].

Manage Image Acquisition Settings

The ***Manage Settings*** modality of Micro-Meta App (Figures 1C and 2C and Extended Data Figure 5) articulates along the following steps:

1) After selecting a 4DN-BINA-OME documentation tier (Extended Data Figure 4A)[2–4] and launching the App in the ***Manage Settings*** modality (Extended Data Figure 5A), the user selects an available *Microscope.JSON* file from the local file system or a suitable repository (Extended Data Figure 5B), selects an available image dataset to be annotated (Extended Data Figure 5C) and either creates a new *Settings.JSON* file or opens an existing file to edit (Extended Data Figure 5D). The integration of the BioFormats API[11] as part of Micro-Meta App permits the App to interpret image file headers, extract available metadata and populate Instrument-specific, OME-compatible, tier-appropriate metadata fields to facilitate metadata annotation.

2) As a result of the previous step, a diagrammatic representation of *Image Acquisition Settings* is displayed to the user and components or fields containing missing metadata values are highlighted in red to solicit the user's attention (Extended Data Figure 5E). After attending to all missing values, the user can then produce a validated 4DN-BINA-OME-compatible[2–4] and tier-appropriate *Settings.JSON* file, which coupled with the corresponding *Microscope.JSON* file can be associated with the relevant image datasets in the local file system or on an appropriate repository, such as the 4DN Data Portal[8,12]. The Manage Settings section of the App consists of four types of user interaction interfaces:

i) Simple buttons with associated tabbed data entry forms, such as those that allow inspection, editing or entry of general information about the image Pixel structure (Extended Data Figure 5E, [1.1 and [1.2]).

ii) Interface for the selection of one of the available hardware components, addition to the *Settings.JSON* file and editing of associated settings metadata fields. This type of user interface is used for *Edit Objective Settings* (Extended Data Figure 5E, [3.1], [3.2] and [3.3]) and is also used for *Edit Imaging Environment*, *Edit Microscope Table Settings*, *Edit Microscope Stand Settings* and *Edit Sample Positioning Settings*.

iii) Specialized Plane-management interface (Extended Data Figure 5E, [2.1], [2.2] and [2.3]). This interface is used either to inspect and, if necessary, edit the automatically imported *Planes* metadata or to record such metadata in case none was available in the header of the image data file to be annotated.

iv) Specialized Channel-management interface. Special attention was dedicated to the development of the GUI utilized to define the configuration and settings of the Light Path (i.e., Light Source → FilterSet → Detector) associated with each individual Channel (Extended Data Figure 5F and G). To this aim, an intuitive Channel GUI (Extended Data Figure 5F, [4.1] and [4.2]) is organized graphically around a visual representation of the Fluorescence Light Path where users can select among different light sources, filters, and detectors available in the underlying *Microscope.JSON* file and provide the appropriate settings to configure a given Channel. For example, the user would first select a given Light Source among those available (Extended Data Figure 5F, [6.1]), and then enter the appropriate Light Source Settings (Extended Data Figure 5F, [6.2]). The same Channel-specific interface can also be used to manage advanced Light Path features, such as in cases in which a custom-developed microscope has to be described (Extended Data Figure 5G).

3) Once all components have been selected and configured, the Image Acquisition Settings are compiled in a structured *Settings.JSON* file (example available on Zenodo as illustrated in the Data Availability section of Online Methods) [6] and saved either locally or remotely as desired.

SUPPLEMENTARY CASE STUDIES

Utilization at core facilities

In response to significant interest we observed in the community and after beta testing (see Online Methods), microscope custodians at 16 international light microscopy facilities (Extended Data Figure 6 - Table III) volunteered to serve as case studies on the use of Micro-Meta App to document both microscope instrumentation and example published image datasets produced in microscopy platforms[13–32], on the basis of the Core + Basic extension of the 4DN-BINA-OME data model[1,2,4,33]. The microscopes whose hardware was documented using Micro-Meta App comprised advanced custom-built microscopes, widefield microscopes and microscope stands associated with confocal systems, produced by all four major manufacturers. As a further testament of the robustness of the approach, several different major categories of imaging experiments were covered in this case study (Figure 2, Extended Data Figures 7-9 and Supplementary Figures 4-15) including:

1) Three-dimensional visualization of superhydrophobic polymer-nanoparticles (Extended Data Figure 7)[22].
2) Investigation of the three-dimensional structure of cerebral organoids after infiltration with Zika virus infected monocytes (Extended Data Figure 8)[19].

3) Immunofluorescence imaging of the three-dimensional distribution of HIV-1 retroviral particles in the nucleus of infected human cells (Figure 2 and Extended Data Figure 9; example *Microscope-* and *Settings.JSON* files for this use case are available on Zenodo as illustrated in the Data Availability section of Online Methods)[6].
4) Immunofluorescence imaging of cryosection of Mouse kidney (Supplementary Figure 4).
5) Three-dimensional immunofluorescence imaging of the phagocytic activity of human rhinovirus 16 (HRV16) infected macrophages (Supplementary Figure 7)[32].
6) Live-cell imaging of *N. benthamiana* leaves cells-derived protoplasts transiently expressing YFP-tagged *P. chromatophora* proteins (Supplementary Figure 9)[34].
7) Single-particle tracking of Halo-tagged PCNA in Lox cells (Supplementary Figure 11)[18].
8) Live-cell imaging of bacterial cells expressing PopZ tagged with super-folder-GFP (Supplementary Figure 14)[23].
9) Transmitted light brightfield visualization of swimming spermatocytes (Supplementary Figure 15)[30,31].

In the case of commercial microscopes and given the type of experimental question and imaging modality, the most appropriate reporting tier-level was 4DN-BINA-OME Tier 2 (Figure 1)[1,2,33]. The exception was represented by one case in which no quantitative analysis was necessary, for which Tier 1 was sufficient (Extended Data Figure 7). On the other hand, two custom-built systems were documented at Tier 3 (Figure 2, and Extended Data Figure 9 and Supplementary Figure 15), again as sanctioned by the 4DN-BINA specifications (Figure 1)[1,2,33].

Teaching with Micro-Meta App

Two major teaching use cases have been explored in the context of the Foundations in Biomedical Science (BBS 614) course[35] administered to first-year students at the Graduate School of Biomedical Sciences at the University of Massachusetts Medical school: 1) Micro-Meta App was used for students to work on specific problem sets; 2) Micro-Meta App was used for self-driven exploration of microscope components, functions and imaging modalities.

In both cases, it is advisable to create specific teaching *Microscope.JSON* files that students can load and work on. Specifically, the features and complexity of these teaching *Microscope.JSON* files need to be aligned with course level and content by choosing the most appropriate tier-level among those available and, if necessary, by structuring the file without adhering to any one specific tier [1,2,33]. For example, a problem set might be assigned that focuses on choosing the most appropriate filter set for a given imaging experiment. Specifically, students are instructed to choose an appropriate light source and then specify each filter to be associated with the filter set. In this case, the depth of information associated with Tier 3 might be needed for the filters and a short list of possible light sources might be provided for the students to choose from (e.g., laser combiner with the wrong laser lines for the experiment and broadband source). At the same time the rest of the *Microscope.JSON* file could be kept at a very basic level to reduce grading complexity. In another example, a course might start on Day 1 with a *Microscope.JSON* file that only has a few components at Tier 1. On subsequent days, more components might be added, and the tier-level might be raised up to Tier 3 depending on the specific course teaching goals.

SUPPLEMENTARY VIDEOS

Supplementary Video 1: Micro-Meta App-an introduction

This video was used as an introduction to the use of the Micro-Meta App that was utilized to instruct Graduate Students at the University of Massachusetts Chen Medical School[35]. Images labeled with (*) and (**) at minutes 0:16 - 0:33 of Supplementary Video 1 are used with permission from the author from Figures 5 and 6 of Smith et al., 2015[36]. The video is also publicly available at: https://vimeo.com/manage/videos/604291798.

SUPPLEMENTARY FIGURES

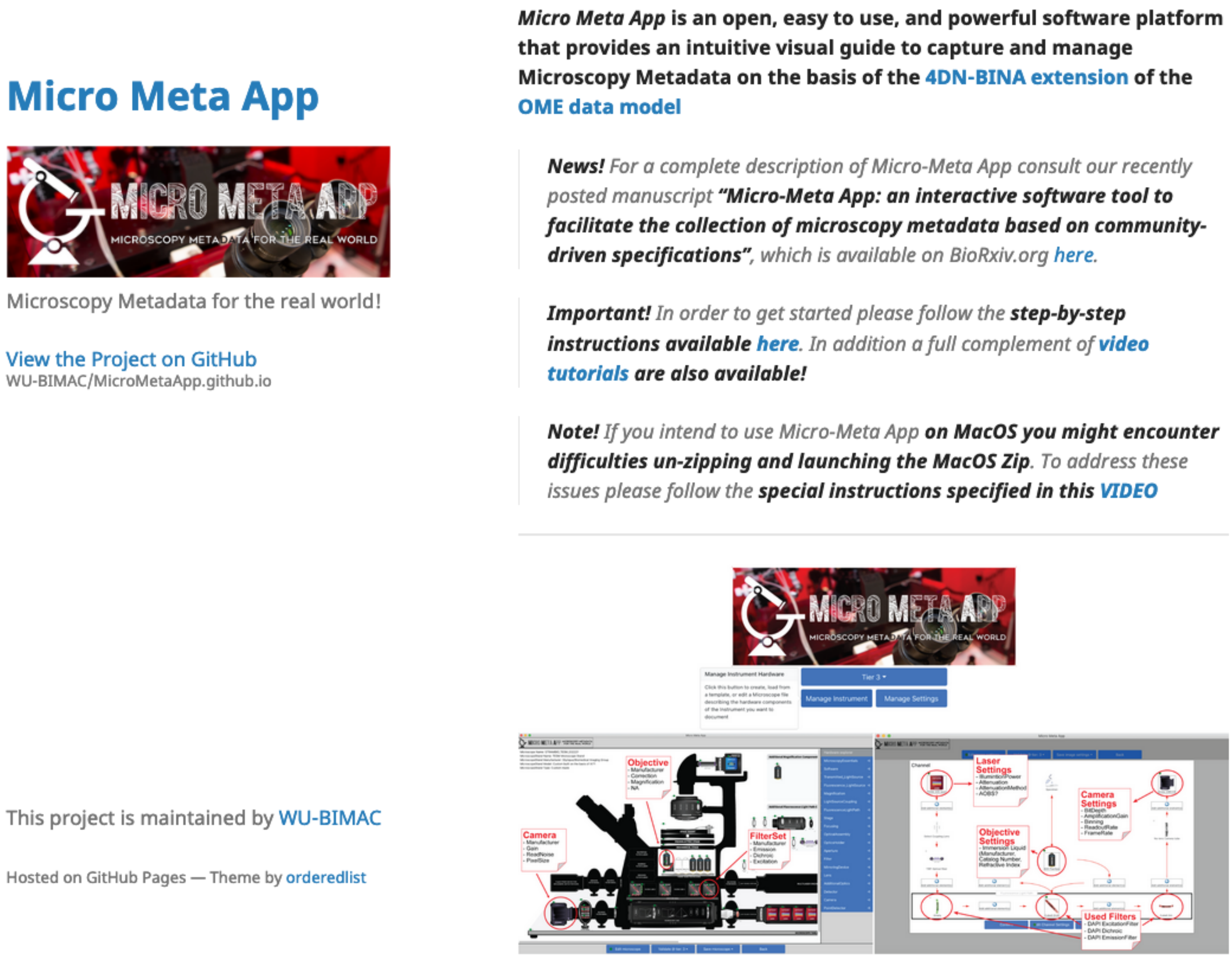

Supplementary Figure 1 | Micro-Meta App website. The Micro-Meta App website is available at https://wu-bimac.github.io/MicroMetaApp.github.io/ and was developed to promote outreach and adoption.

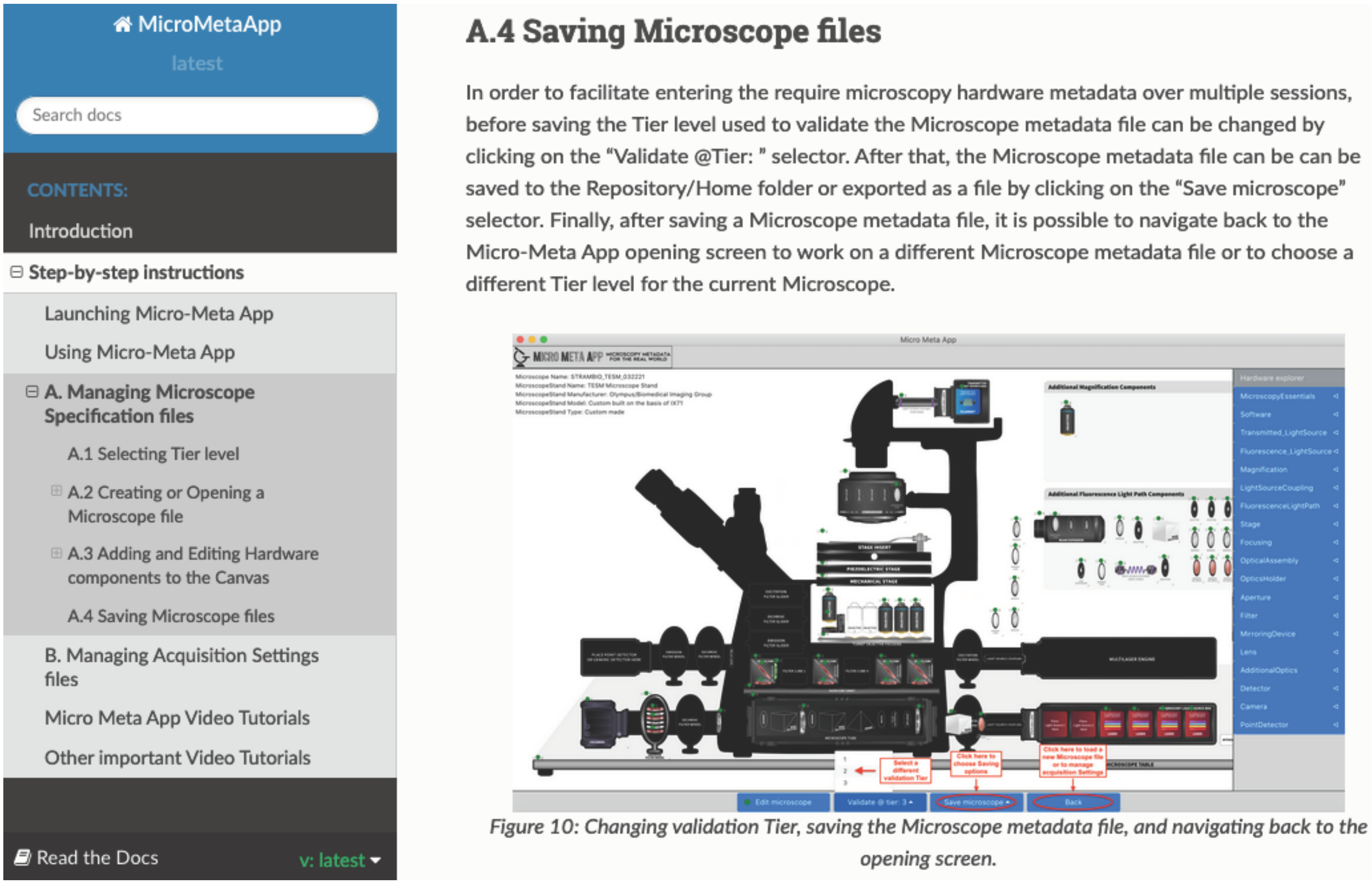

Supplementary Figure 2 | Micro-Meta App Read the Docs documentation and tutorial site. The Micro-Meta App ReadtheDocs documentation is available at https://micrometaapp-docs.readthedocs.io/en/latest/index.html. The site contains step-by-step instructions and video tutorials that explain how to use the App.

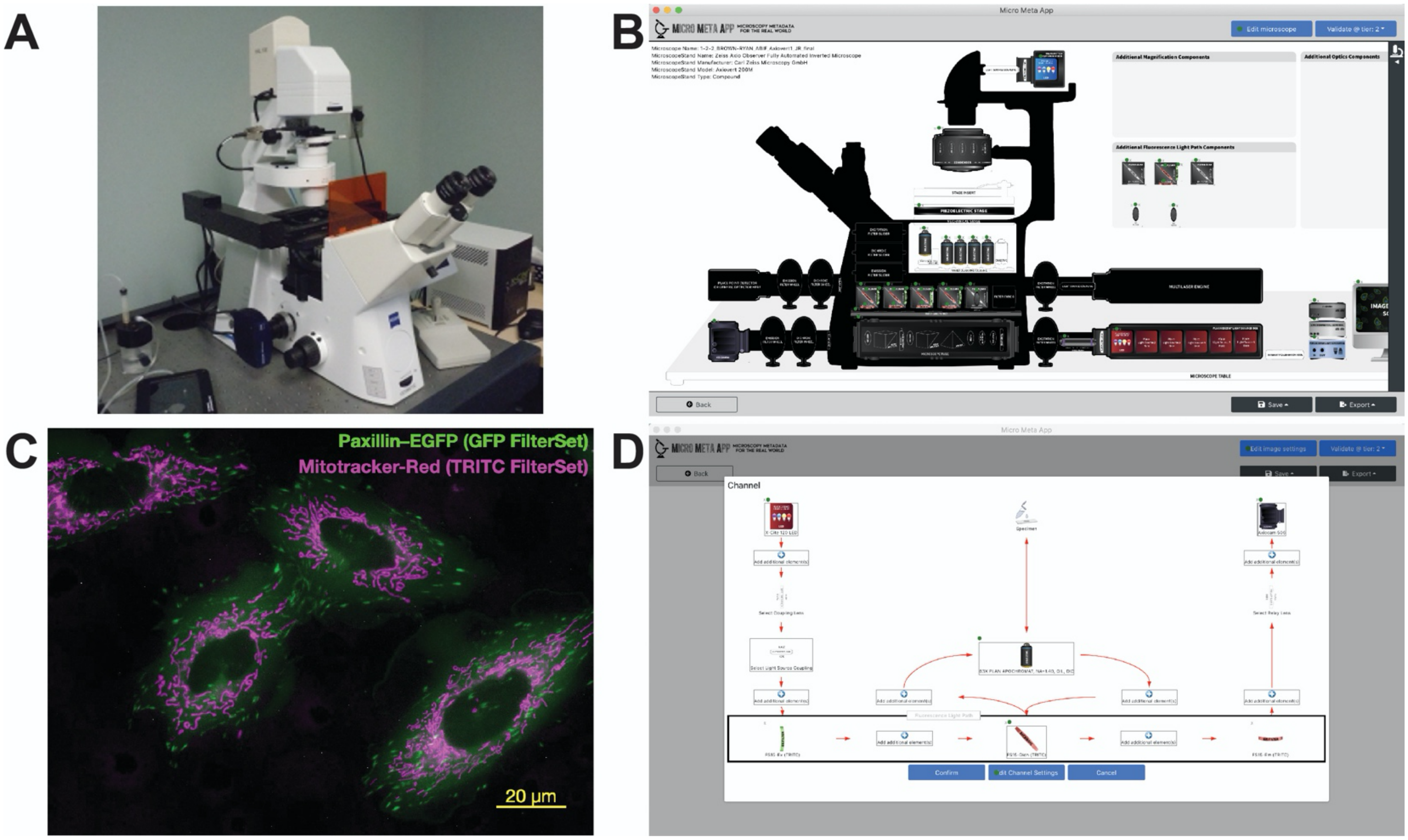

Supplementary Figure 3 | Micro-Meta App was utilized at the Advanced BioImaging Facility of McGill University to document a Zeiss Axio Observer inverted, epifluorescence microscope and the settings that were applied to the microscope to acquire specific example datasets. Illustrated here is the use of Micro-Meta App for Tier 2 *(Advanced Quantification and/or Live cell Imaging)*, 4DN-BINA-OME-specified[2,4] documentation of: **(A-B)** the ***Zeiss Axio Observer (Axiovert 200M)*** inverted, epifluorescence microscope owned by the Advanced BioImaging Facility (ABIF) of McGill University (Extended Data Figure 6 - Table III); and **(C-D)** the settings that were applied to the microscope for the acquisition of example published image datasets[16]. **A)** Picture of the indicated microscope. **B)** Micro-Meta App generated schematic representation of the indicated microscope. **C)** Chinese Hamster Ovary K1 (CHO-K1) cells stably expressing paxillin–EGFP to visualize the cytoplasm and adhesions were seeded onto fibronectin-coated glass coverslips, allowed to adhere and grow overnight, and stained with MitoTracker Red to visualize mitochondrial morphology. Images for Paxillin–EGFP (GFP channel, visualized in green) and MitoTracker™ Red (TRITC channel, visualized in magenta) were acquired every minute and 6 min, respectively, using the indicated microscope (Panels A and B) with Diffuse Light Delivery (DLD; 0.0093 mW×60,000 ms) illumination. Displayed is a representative image from time zero[16]. **D)** Micro-Meta App generated schematic representation of the light path associated with the ***TRITC Channel*** utilized for the acquisition of the image in Panel C. To demonstrate the functionality and usability of the App and to assess the feasibility of the overall microscopy documentation approach, Micro-Meta App was tested independently at 16 sites (Extended Data Figures 7-9 and Supplementary Figures 3-15).

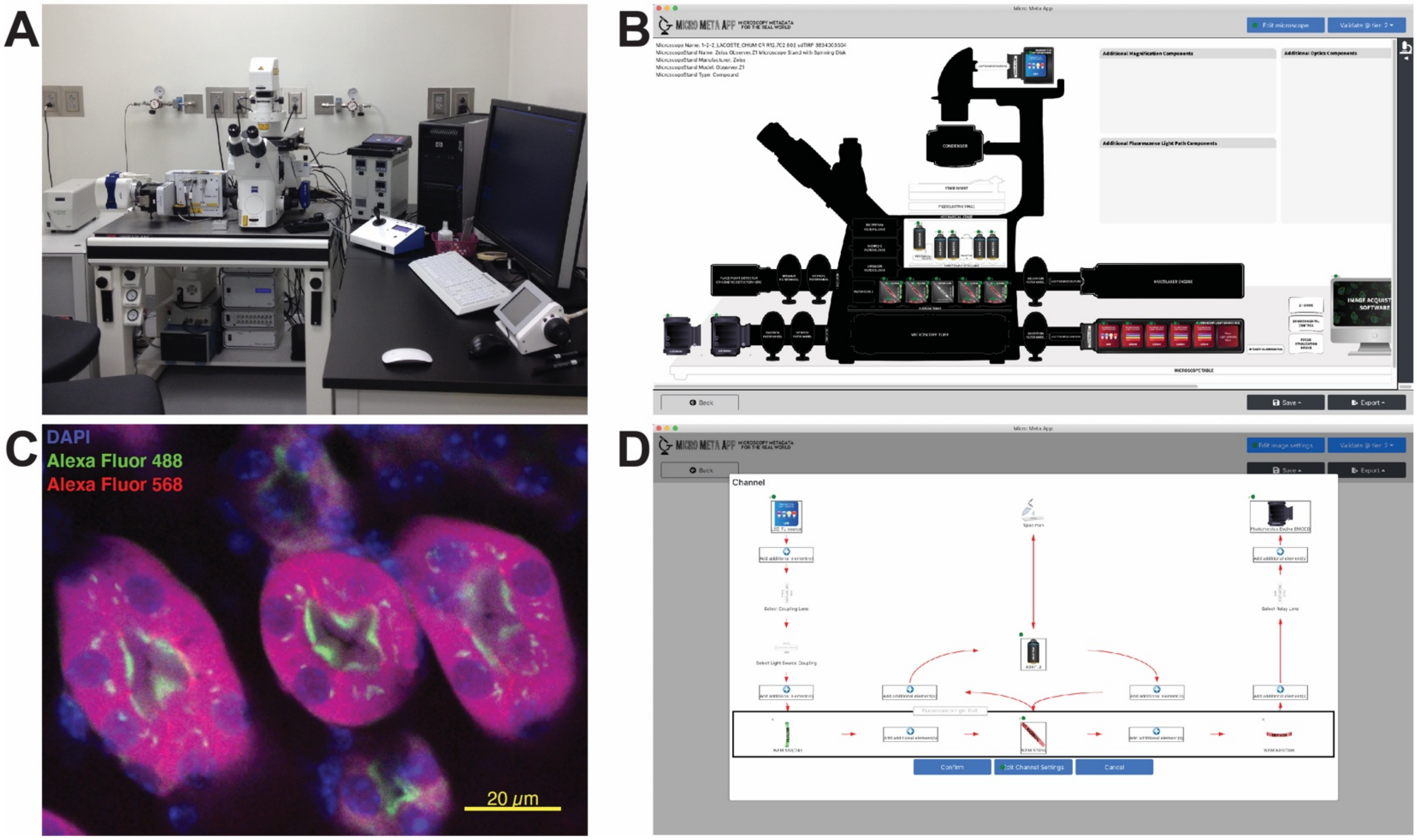

Supplementary Figure 4 | Micro-Meta App was utilized by MIA Cellavie Inc. at the "Imagerie Cellulaire" core facility of the University of Montreal to document a Zeiss Observer.Z1 inverted, epifluorescence microscope with Spinning Disk and the settings that were applied to the microscope to acquire specific example datasets. Illustrated here is the use of Micro-Meta App for Tier 2 *(Advanced Quantification and/or Live cell Imaging)*, 4DN-BINA-OME-specified[2] documentation of: **(A-B)** the ***Zeiss Observer.Z1*** inverted, epifluorescence microscope with Spinning Disk[37] owned by the "Imagerie Cellulaire" core facility of the CR CHUM (Centre de recherche du Centre Hospitalier de l'Université de Montréal), and whose calibration and performance quality control is managed by MIA Cellavie Inc. (Extended Data Figure 6 - Table III); and **(C-D)** the settings that were applied to the microscope for the acquisition of the indicated example image datasets. **A)** Picture of the indicated microscope. **B)** Micro-Meta App generated schematic representation of the indicated microscope. **C)** Displayed is a representative single-plane image of a 16 µm cryosection of Mouse kidney stained with DAPI (Blue channel, displayed in blue), WGA-AlexaFluor488 (Green channel, displayed in green), and Phalloidin-Alexa Fluor 568 (Red channel, displayed in red), and mounted in Gelvatol mounting medium. The displayed image was obtained using the indicated microscope in Panels A and B. **D)** Micro-Meta App generated schematic representation of the light path associated with the ***Red Channel*** utilized for the acquisition of the image in Panel C. To demonstrate the functionality and usability of the App and to assess the feasibility of the overall microscopy documentation approach, Micro-Meta App was tested independently at 16 sites (Extended Data Figures 7-9 and Supplementary Figures 3-15).

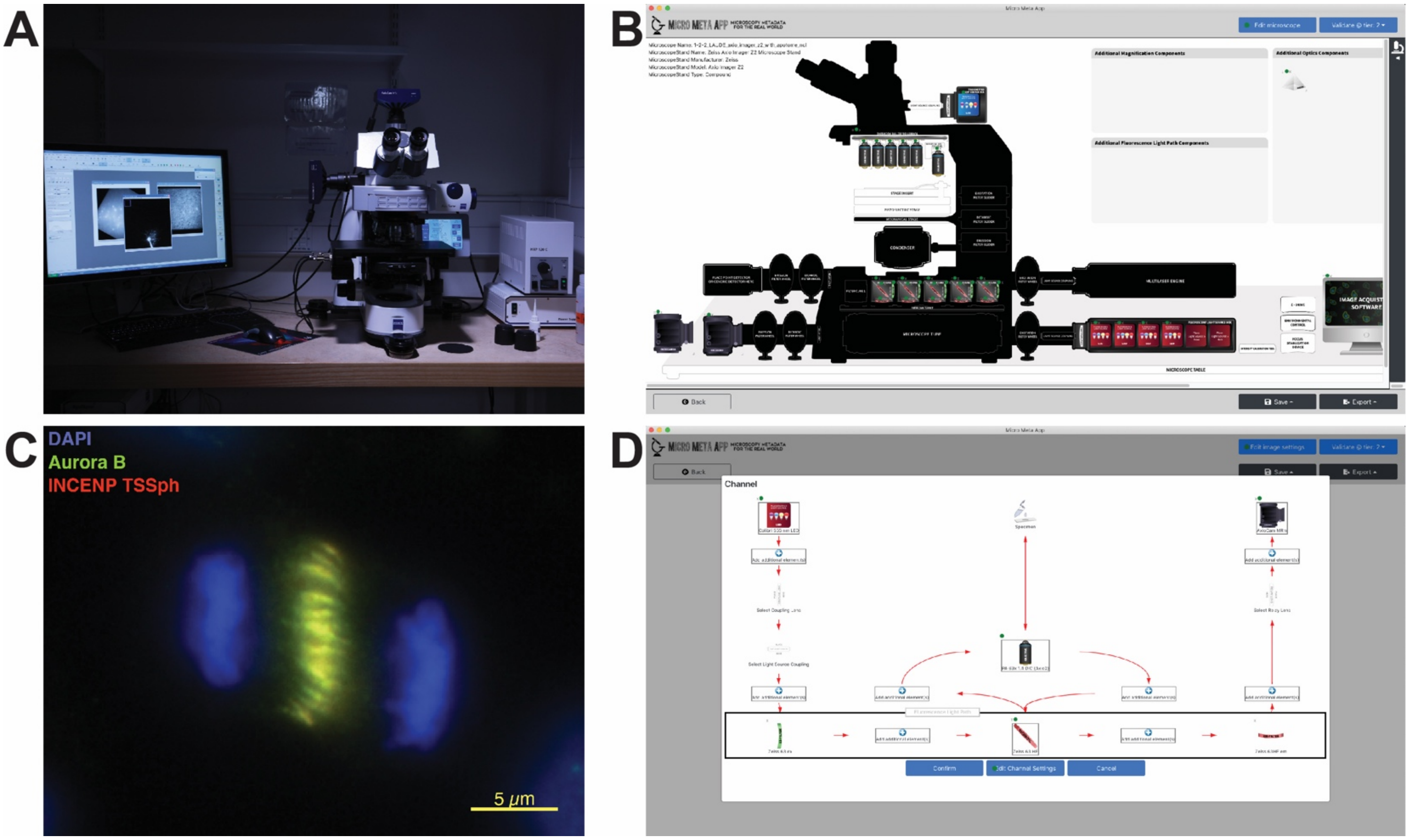

Supplementary Figure 5 | Micro-Meta App was utilized by the Bioimaging Unit of the University of Newcastle to document a Zeiss Axio Imager Z2 upright epifluorescence microscope and the settings that were applied to the microscope to acquire specific example datasets. Illustrated here is the use of Micro-Meta App for Tier 2 *(Advanced Quantification and/or Live cell Imaging)*, 4DN-BINA-OME-specified[2,4] documentation of: **(A-B)** the ***Zeiss Axio Imager Z2*** upright epifluorescence microscope with Apotome owned by the Bioimaging Unit of the University of Newcastle (Extended Data Figure 6 - Table III); and **(C-D)** the settings that were applied to the microscope for the acquisition of example published image datasets[21]. **A)** Picture of the indicated microscope. **B)** Micro-Meta App generated schematic representation of the indicated microscope. **C)** Mitotic HeLa cells were labeled for: DNA (DAPI DNA stain, DAPI channel, Zeiss 49 Filter set, visualized in blue), Aurora B (primary sheep anti-Aurora B polyclonal antibody, followed by donkey anti-sheep Alexa Fluor 488 secondary; GFP channel, Zeiss 38HE Filter set, displayed in green), and INCENP TSSph (primary rabbit anti-INCENP-TSSph polyclonal antibody, followed by donkey anti-rabbit Alexa Fluor 488 secondary; Red channel, Zeiss 63HE Filter set, displayed in red). Images were captured by widefield fluorescence microscopy using the microscope displayed in A & B equipped with a Colibri2 LED excitation light source, a Zeiss Plan Apo 63x/ 1.4NA objective, and a AxioCam MRmV3 camera. The displayed image was obtained using the indicated microscope (Panels A and B)[21]. **D)** Micro-Meta App generated schematic representation of the light path associated with the ***INCENP-TSSph (Alexa Fluor 594) Channel*** utilized for the acquisition of the image in Panel C. To demonstrate the functionality and usability of the App and to assess the feasibility of the overall microscopy documentation approach, Micro-Meta App was tested independently at 16 sites (Extended Data Figures 7-9 and Supplementary Figures 3-15).

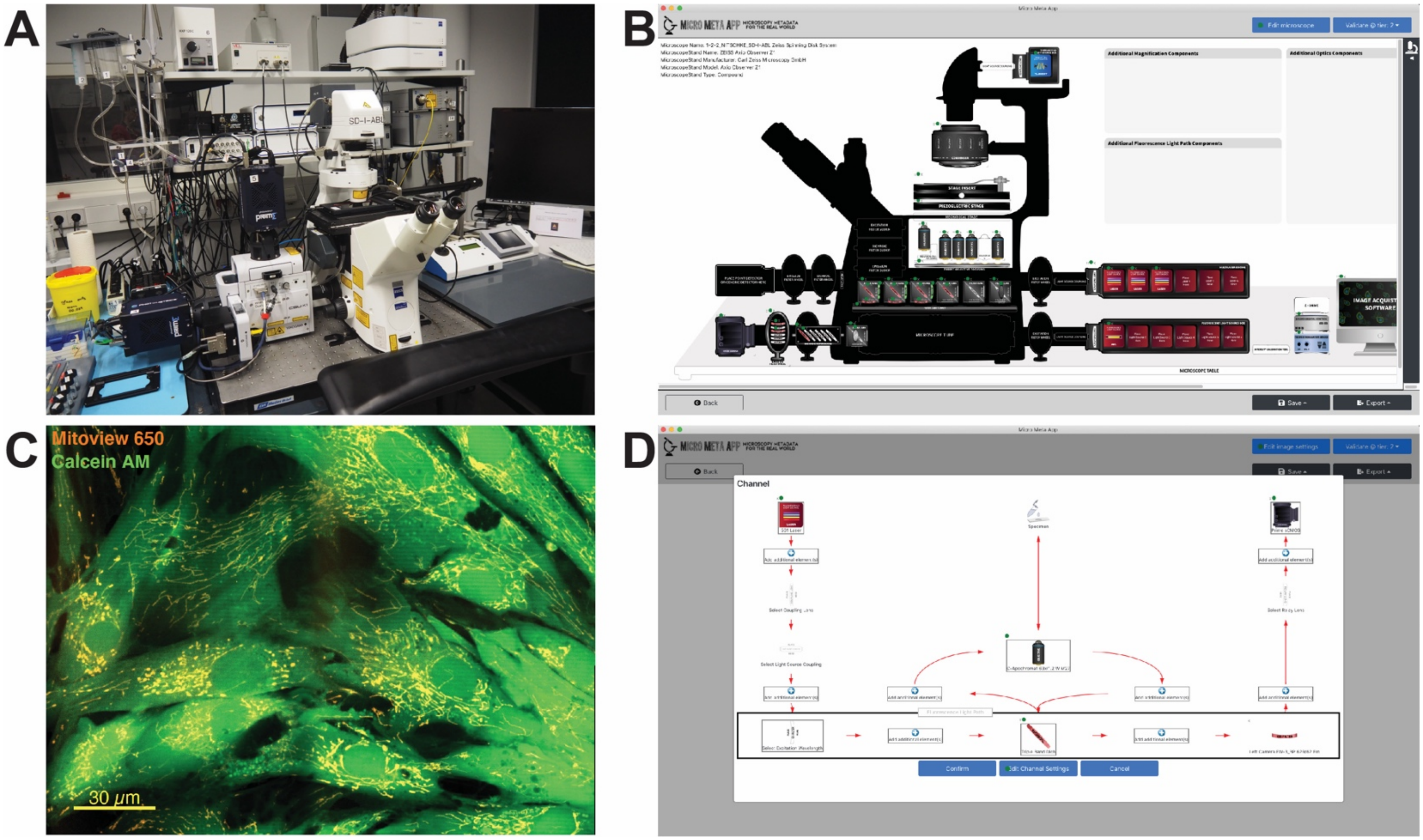

Supplementary Figure 6 | Micro-Meta App was utilized by the Life Imaging Center of the University of Freiburg to document a Zeiss Axio Observer Z1 inverted, epifluorescence microscope and the settings that were applied to the microscope to acquire specific example datasets. Illustrated here is the use of Micro-Meta App for <u>Tier 2</u> *(Advanced Quantification and/or Live cell Imaging)*, 4DN-BINA-OME-specified[2] documentation of: **(A-B)** the ***Zeiss Axio Observer Z1*** inverted, epifluorescence microscope owned by the Life Imaging Center (LIC) Centre for Integrative Signaling Analysis (CISA) of the University of Freiburg (Extended Data Figure 6 - Table III); and **(C-D)** the settings that were applied to the microscope for the acquisition of example published image datasets[29]. **A)** Picture of the indicated microscope. **B)** Micro-Meta App generated schematic representation of the indicated microscope. **C)** Microscopy of live human skin fibroblasts from Glycogen Storage Disease (GSD) patients. After growth under standard conditions (humidified 5% CO2 incubator at 37°C) for 3–5 days, cells were trypsinized and 1000 cells were seeded in glass-bottom cell culture dishes. Cells were grown overnight under standard conditions and live cell staining was applied using Calcein™ AM for cell body (Ch 1 - GFP channel, displayed in green), and MitoView™ 650 for mitochondria (Ch 2 - AF555 channel, displayed in red). A representative overlay image is presented[29]. **D)** Micro-Meta App generated schematic representation of the light path associated with the ***Ch2 – AF555 Channel*** utilized for the acquisition of the image in Panel C. To demonstrate the functionality and usability of the App and to assess the feasibility of the overall microscopy documentation approach, Micro-Meta App was tested independently at 16 sites (Extended Data Figures 7-9 and Supplementary Figures 3-15).

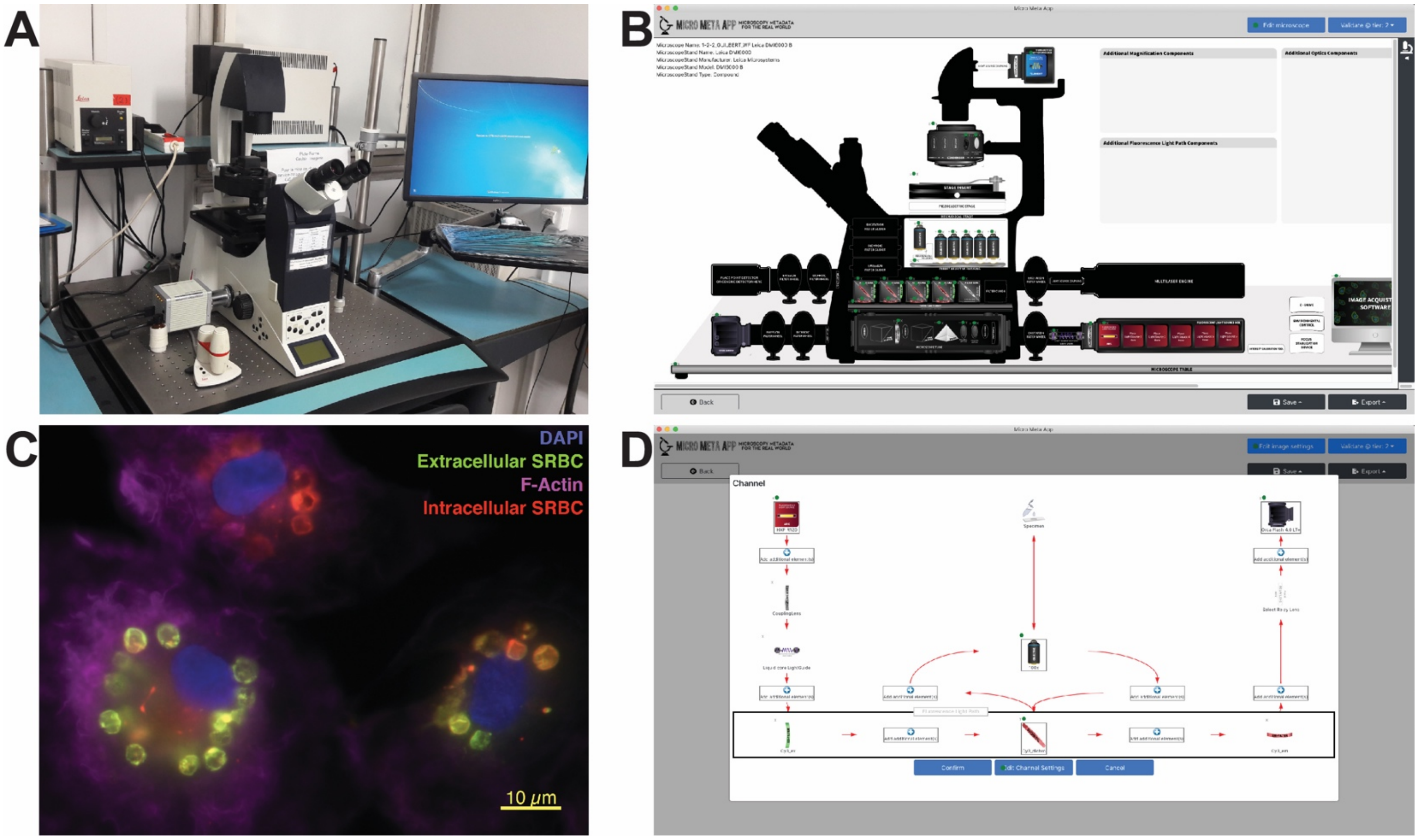

Supplementary Figure 7 | Micro-Meta App was utilized by the IMAG'IC Confocal Microscopy Facility of the Institut Cochin, at the Université de Paris to document a Leica Microsystems DMI6000 inverted epifluorescence microscope and the settings that were applied to the microscope to acquire specific example datasets. Illustrated here is the use of Micro-Meta App for Tier 2 *(Advanced Quantification and/or Live cell Imaging)*, 4DN-BINA-OME-specified[2,4] documentation of: **(A-B)** the ***Leica Microsystems DMI6000*** inverted epifluorescence microscope owned by the IMAG'IC Confocal Microscopy Facility of the Institut Cochin, at the Université de Paris (Extended Data Figure 6 - Table III); and **(C-D)** the settings that were applied to the microscope for the acquisition of example published image datasets[32]. **A)** Picture of the indicated microscope. **B)** Micro-Meta App generated schematic representation of the indicated microscope. **C)** Primary human macrophages were treated with human rhinovirus 16 (HRV16) for one hour, washed, and allowed to rest overnight at 37°C. The cells were then incubated at 37°C for 60 min with rabbit IgG-opsonized Sheep Red Blood Cells (SRBCs) to induce phagocytosis. At each time point, macrophages were fixed and labeled with Alexa Fluor 488-labeled F (ab') anti-rabbit IgG to detect the external SRBCs (GFP channel, visualized in green), then permeabilized and labeled with Cy5-labelled F (ab') anti-rabbit IgG to detect the internal SRBCs (CY5 channel, visualized in red), with Alexa Fluor 546-conjugated phalloidin to detect F-actin (CY3 Channel, visualized in magenta), and DAPI to stain the nuclei (DAPI channel, visualized blue). The displayed image was obtained using the indicated microscope (Panels A and B)[32]. **D)** Micro-Meta App generated schematic representation of the light path associated with the ***F-Actin (Phalloidin-AF546) Channel*** utilized for the acquisition of the image in Panel C. To demonstrate the functionality and usability of the App and to assess the feasibility of the overall microscopy documentation approach, Micro-Meta App was tested independently at 16 sites (Extended Data Figures 7-9 and Supplementary Figures 3-15).

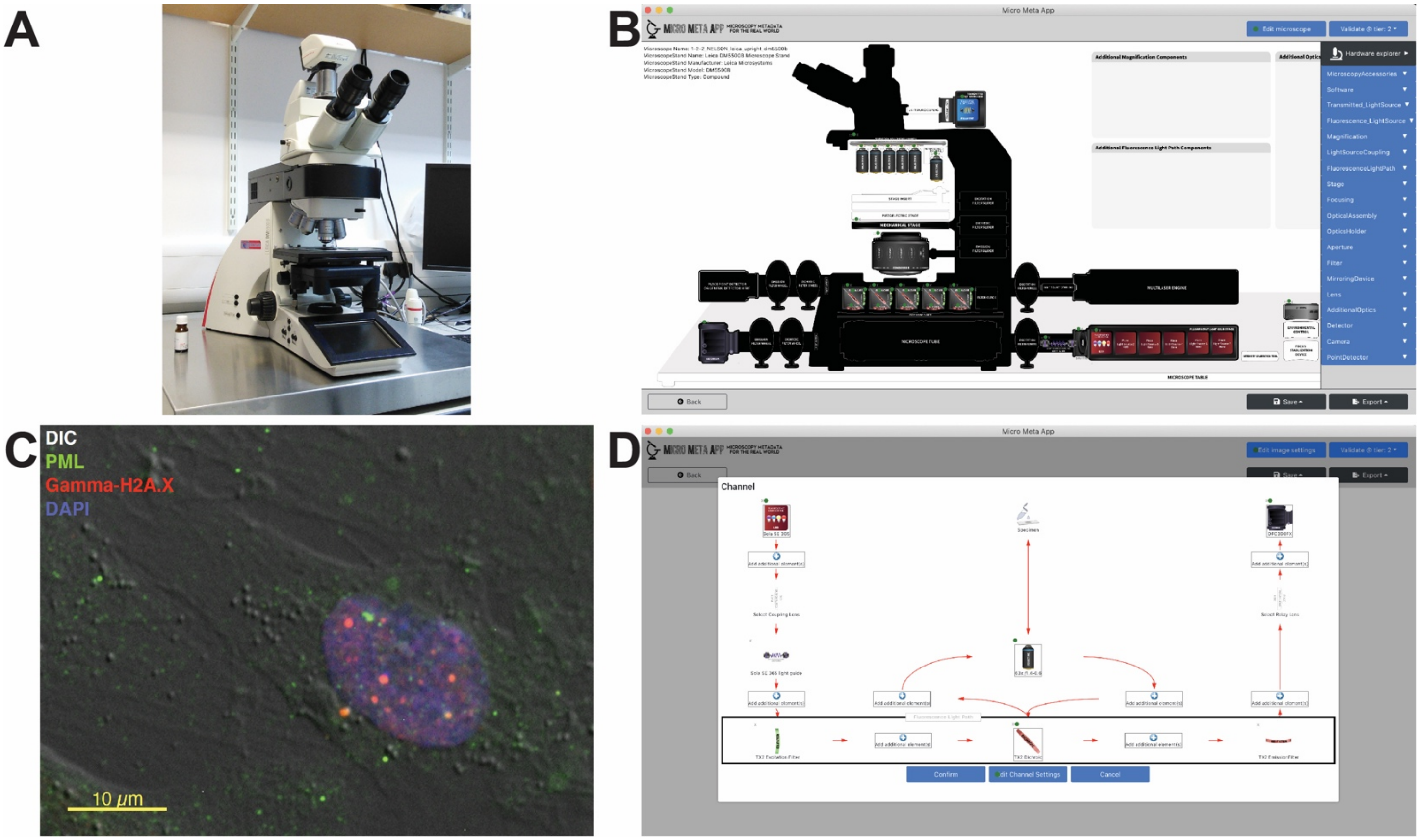

Supplementary Figure 8 | Micro-Meta App was utilized by the Bioimaging Unit at the Edwardson Building on the Campus for Ageing and Vitality of the University of Newcastle to document a Leica Microsystems DM5500B upright, epifluorescence microscope and the settings that were applied to the microscope to acquire specific example datasets. Illustrated here is the use of Micro-Meta App for Tier 2 *(Advanced Quantification and/or Live cell Imaging)*, 4DN-BINA-OME-specified[2] documentation of: **(A-B)** the ***Leica Microsystems DM5500B*** upright, epifluorescence microscope owned by the Bioimaging Unit and located in the Edwardson Building on the Campus for Ageing and Vitality of the University of Newcastle (Extended Data Figure 6 - Table III); and **(C-D)** the settings that were applied to the microscope for the acquisition of example published image datasets[38]. **A)** Picture of the indicated microscope. **B)** Micro-Meta App generated schematic representation of the indicated microscope. **C)** Immunofluorescence of young human diploid fibroblasts measuring the frequency of colocalization of PML bodies (as measured by staining cells with anti-PML primary antibody, followed by Alexa Fluor 488-conjugated secondary antibody; green) with DNA double-strand breaks (as measured by staining cells with anti-gamma-H2A.X primary antibody, followed by Alexa Fluor 555-conjugated secondary antibody; red), overlaid with a DIC transmission image (grey) and DAPI nuclear counterstain (blue) when grown in the presence or absence of senescent cells for 10 or 20 days. The displayed image was obtained using the indicated microscope (Panels A and B)[38]. **D)** Micro-Meta App generated schematic representation of the light path associated with the ***CY3 (gamma-H2A.X; red) Channel*** utilized for the acquisition of the image in Panel C. To demonstrate the functionality and usability of the App and to assess the feasibility of the overall microscopy documentation approach, Micro-Meta App was tested independently at 16 sites (Extended Data Figures 7-9 and Supplementary Figures 3-15).

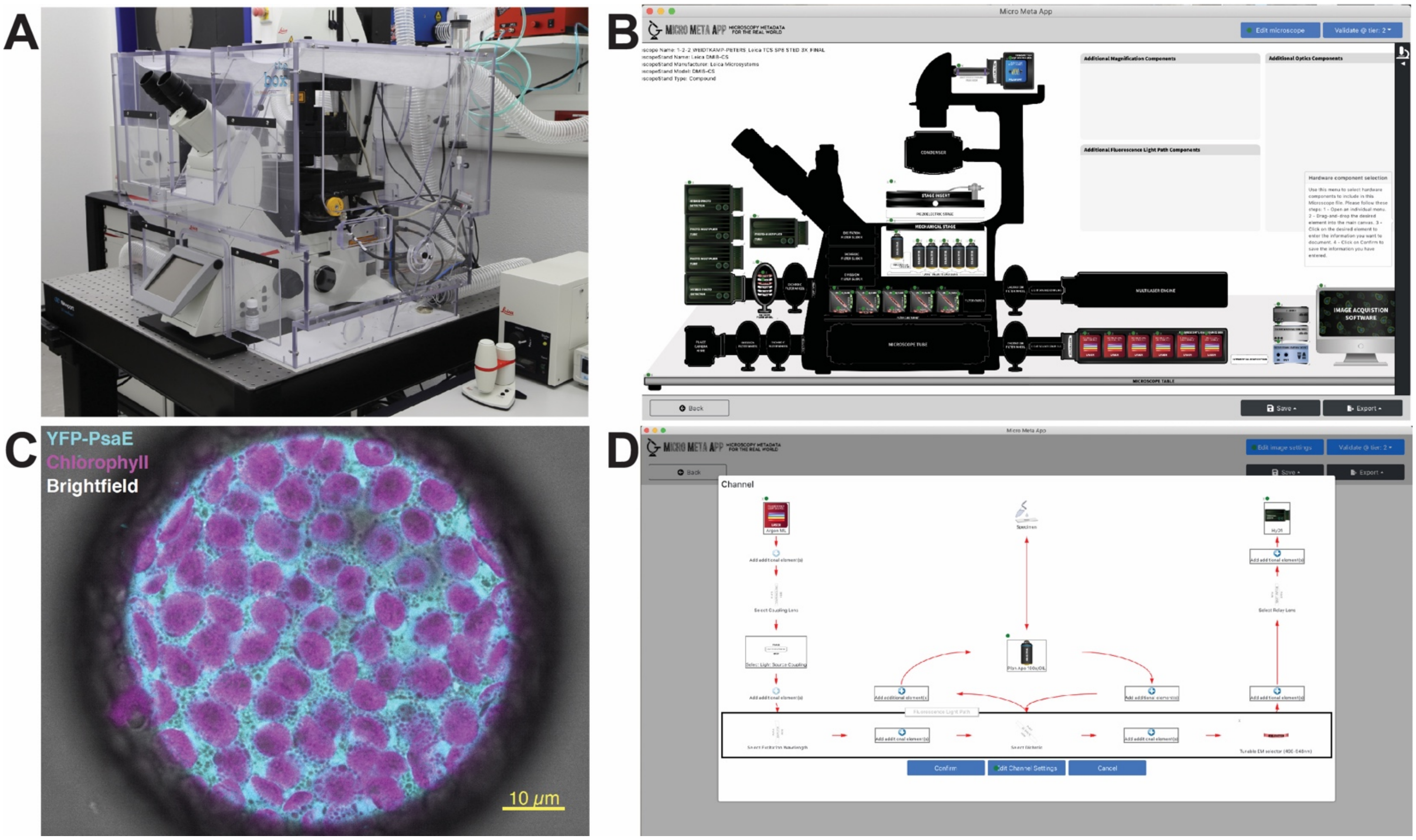

Supplementary Figure 9 | Micro-Meta App was utilized by the Center for Advanced imaging at the Heinrich-Heine-Universität Düsseldorf to document a Leica Microsystems TCS SP8 STED 3X laser scanning confocal microscope system and the settings that were applied to the microscope to acquire specific example datasets. Illustrated here is the use of Micro-Meta App for Tier 2 *(Advanced Quantification and/or Live cell Imaging)*, 4DN-BINA-OME-specified[2,4] documentation of: **(A-B)** the ***Leica Microsystems TCS SP8 STED 3X laser scanning confocal microscope system (with DMI8-CS inverted compound microscope stand)***, owned by the Center for Advanced imaging (CAi) at the School of Mathematics and Natural Sciences of the Heinrich-Heine-Universität Düsseldorf (Extended Data Figure 6 - Table III); and **(C-D)** the settings that were applied to the microscope for the acquisition of example published image datasets[34] (Singer et al., 2017). **A)** Picture of the indicated microscope. **B)** Micro-Meta App generated schematic representation of the indicated microscope. **C)** *N. benthamiana* cells were transformed to transiently express an N-Terminally YPF-tagged variant of the *P. chromatophora* PsaE protein, and prepared to produce protoplasts. Washed protoplasts were subsequently mounted on glass slides and prepared for multi-color fluorescence and transmitted light microscopy observation using the microscope illustrated in Panels A and B, equipped with a Leica HC PL APO 100x/ 1.40 OIL CS2 objective. Shown with permission[34] is a representative image of a protoplast in which the YFP fluorescence channel (*YFP-PsaE*, 488 laser, 498-548nm emission selection; displayed in Cyan) is overlaid with the Chlorophyll autofluorescence channel (*Chlorophyll*, 488 laser, 649-687nm emission selection; displayed in Magenta) and with the transmitted light channel (*Brightfield*; displayed in Grey)[34]. **D)** Micro-Meta App generated schematic representation of the light path associated with ***Channel 0 (YFP-PsaE)*** utilized for the acquisition of the image in Panel C. To demonstrate the functionality and usability of the App and to assess the feasibility of the overall microscopy documentation approach, Micro-Meta App was tested independently at 16 sites (Extended Data Figures 7-9 and Supplementary Figures 3-15).

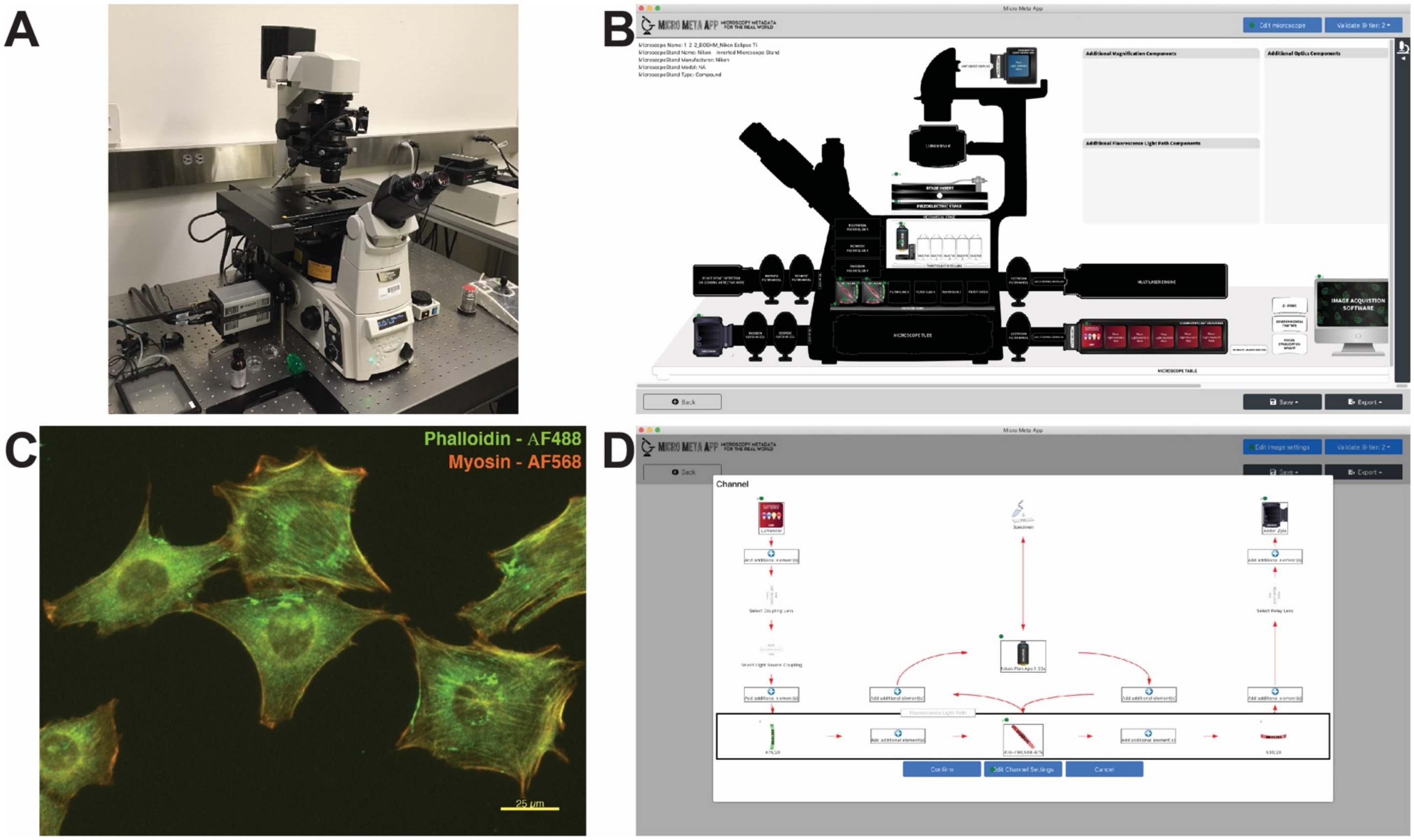

Supplementary Figure 10 | Micro-Meta App was utilized at the Janelia Research Campus of the Howard Hughes Medical Institute to document a Nikon Eclipse Ti inverted, epifluorescence microscope and the settings that were applied to the microscope to acquire specific example datasets. Illustrated here is the use of Micro-Meta App for Tier 2 *(Advanced Quantification and/or Live cell Imaging)*, 4DN-BINA-OME-specified[2] documentation of: **(A-B)** the ***Nikon Eclipse Ti*** inverted, epifluorescence microscope owned by the Advanced Imaging Center (AIC) at the Janelia Research Campus of the Howard Hughes Medical Institute (Extended Data Figure 6 - Table III); and **(C-D)** the settings that were applied to the microscope for the acquisition of example acquired by Ulrike Boehm. **A)** Picture of the indicated microscope. **B)** Micro-Meta App generated schematic representation of the indicated microscope. **C)** COS cells were fixed, permeabilized, blocked, and stained with Alexa Fluor 488-conjugated Phalloidin (488 channel, visualized in green), and anti-Myosin antibodies (visualized with Alexa Fluor 568 conjugated secondary antibody; 568 channel, visualized in magenta). Displayed is a representative single plane image obtained using the indicated microscope (Panels A and B). **D)** Micro-Meta App generated schematic representation of the light path associated with the ***488 Channel*** utilized for the acquisition of the image in Panel C. To demonstrate the functionality and usability of the App and to assess the feasibility of the overall microscopy documentation approach, Micro-Meta App was tested independently at 16 sites (Extended Data Figures 7-9 and Supplementary Figures 3-15).

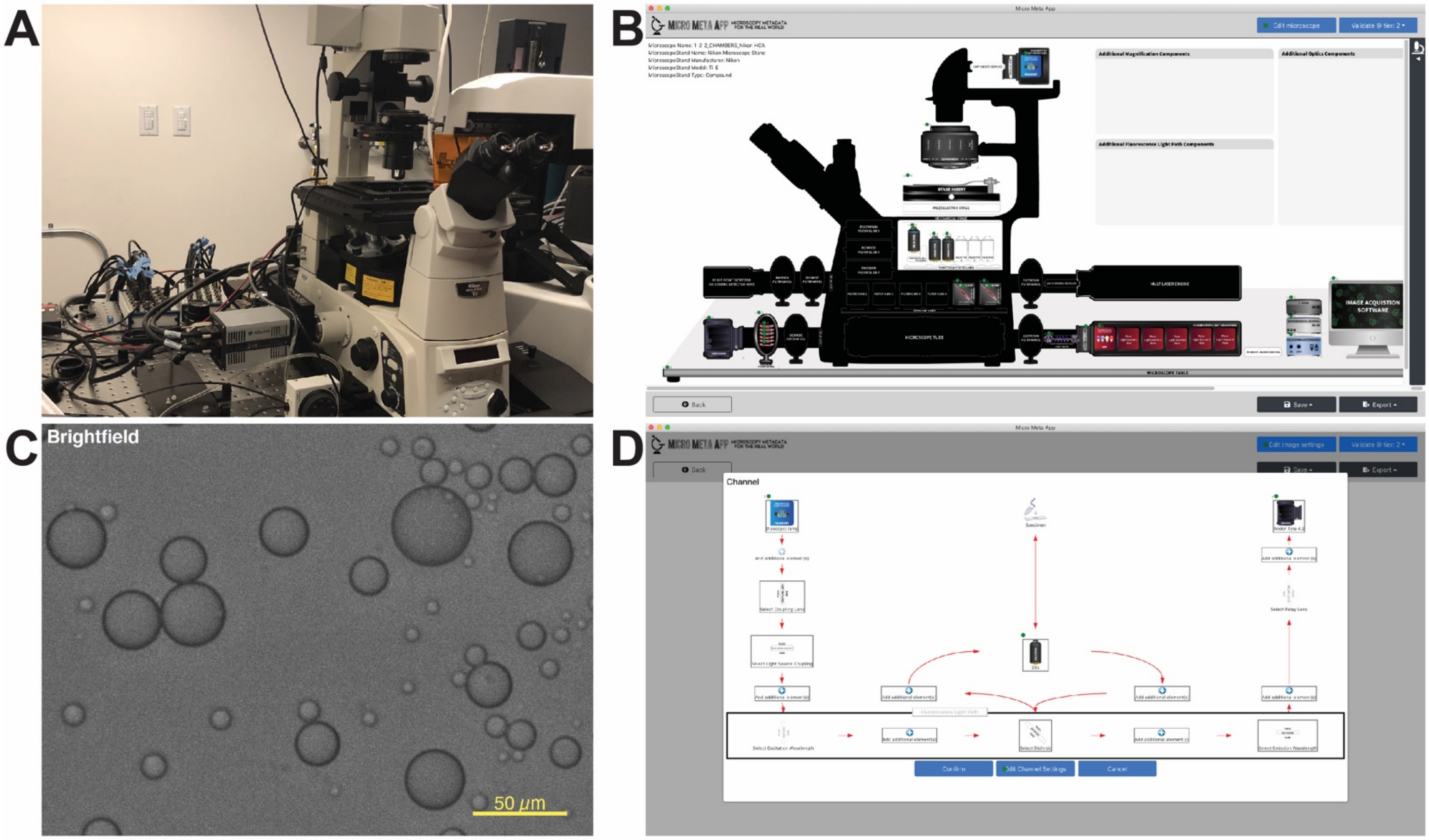

Supplementary Figure 11 | Micro-Meta App was utilized by the Light Microscopy Facility at the Institute for Applied Life Sciences of the University of Massachusetts at Amherst to document a Nikon Eclipse Ti-E inverted epifluorescence microscope and the settings that were applied to the microscope to acquire specific example datasets. Illustrated here is the use of Micro-Meta App for <u>Tier 2</u> *(Advanced Quantification and/or Live cell Imaging)*, 4DN-BINA-OME-specified[2,4] documentation of: **(A-B)** the ***Nikon Eclipse Ti-E*** inverted, epifluorescence microscope owned by the Light Microscopy Facility at the Institute for Applied Life Sciences ((IALS-LIF) of the University of Massachusetts at Amherst (Extended Data Figure 6 - Table III); and **(C-D)** the settings that were applied to the microscope for the acquisition of example published image datasets[17]. **A)** Picture of the indicated microscope. **B)** Micro-Meta App generated schematic representation of the indicated microscope. **C)** Diascopic image of amine-functionalized vesicular assemblies before exposure to a component that results in bursting behavior in the study of new drug delivery macromolecular assemblies. This image is part of a time series (10 us exposure in 12-bit mode) using the 20x objective and is representative of the type of data collected to measure bursting behavior and dynamics. The displayed image was obtained using the indicated microscope (Panels A and B)[17]. **D)** Micro-Meta App generated schematic representation of the light path associated with the ***Brightfield Channel*** utilized for the acquisition of the image in Panel C. To demonstrate the functionality and usability of the App and to assess the feasibility of the overall microscopy documentation approach, Micro-Meta App was tested independently at 16 sites (Extended Data Figures 7-9 and Supplementary Figures 3-15).

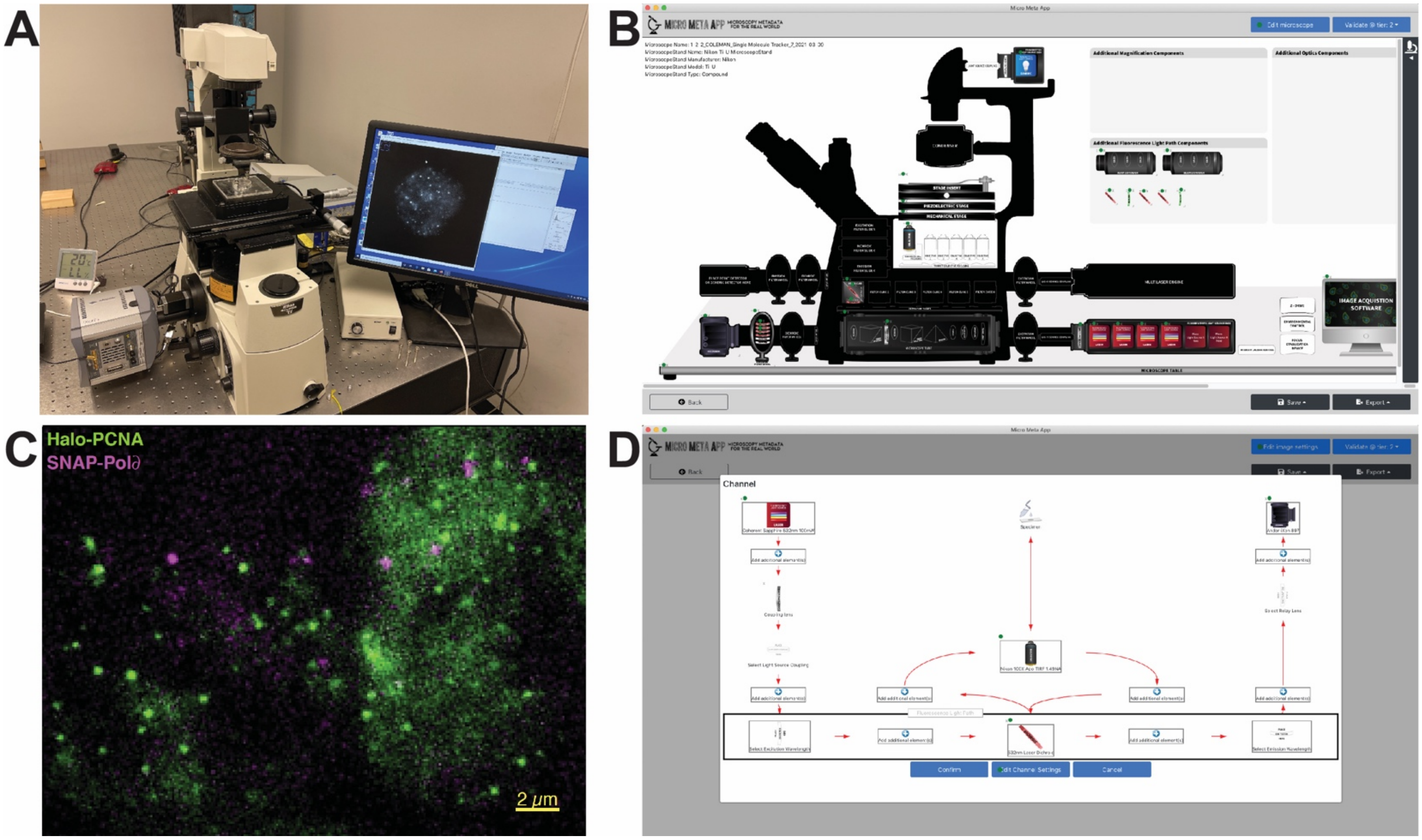

Supplementary Figure 12 | Micro-Meta App was utilized by the Coleman laboratory at the Anatomy and Structural Biology Department of The Albert Einstein College of Medicine to document a custom-built TIRF HILO Epifluorescence light Microscope and the settings that were applied to the microscope to acquire specific example datasets. Illustrated here is the use of Micro-Meta App for Tier 2 *(Advanced Quantification and/or Live cell Imaging)*, 4DN-BINA-OME-specified[2] documentation of: **(A-B)** the custom build ***TIRF HILO Epifluorescence light Microscope (THEM; based on Nikon Eclipse Ti)*** developed, built and owned by the Coleman laboratory at the Anatomy and Structural Biology Department of The Albert Einstein College of Medicine (Extended Data Figure 6 - Table III); and **(C-D)** the settings that were applied to the microscope for the acquisition of example published image datasets[18]. **A)** Picture of the indicated microscope. **B)** Micro-Meta App generated schematic representation of the indicated microscope. **C)** LOX cells stably expressing Halo-PCNA and SNAP-Pol∂ were incubated at 37 C in selective media containing Doxycycline. Immediately prior to imaging, cells were incubated at 37 C with JF549-HTL (Janelia Labs) and SNAP-Cell647-SiR (New England Biolabs), washed and imaged at room temperature. Cells displaying a punctate pattern for PCNA were continuously illuminated using 532nm (13 W/cm2, Coherent) and 640 nm (9.5 W/cm2, Coherent) lasers for JF549-HTL and SNAP-Cell 647-SiR imaging respectively. Two dimensional time-lapse images of single molecules were acquired using the indicated microscope (Panels A and B)[18]. Sequential dual color images of live cell nuclei were acquired for 22 minutes using 500 ms exposures on an EMCCD camera (iXon, Andor) with continuously alternating between the HALO-PCNA (labeled with JF549-HTL, displayed in red) and the SNAP-Pol∂ (labeled with SNAP-Cell 647-SiR, displayed in magenta) channels. **D)** Micro-Meta App generated schematic representation of the light path associated with the ***HALO-PCNA (JF549-HTL) Channel*** utilized for the acquisition of the image in Panel C. To demonstrate the functionality and usability of the App and to assess the feasibility of the overall microscopy documentation approach, Micro-Meta App was tested independently at 16 sites (Extended Data Figures 7-9 and Supplementary Figures 3-15).

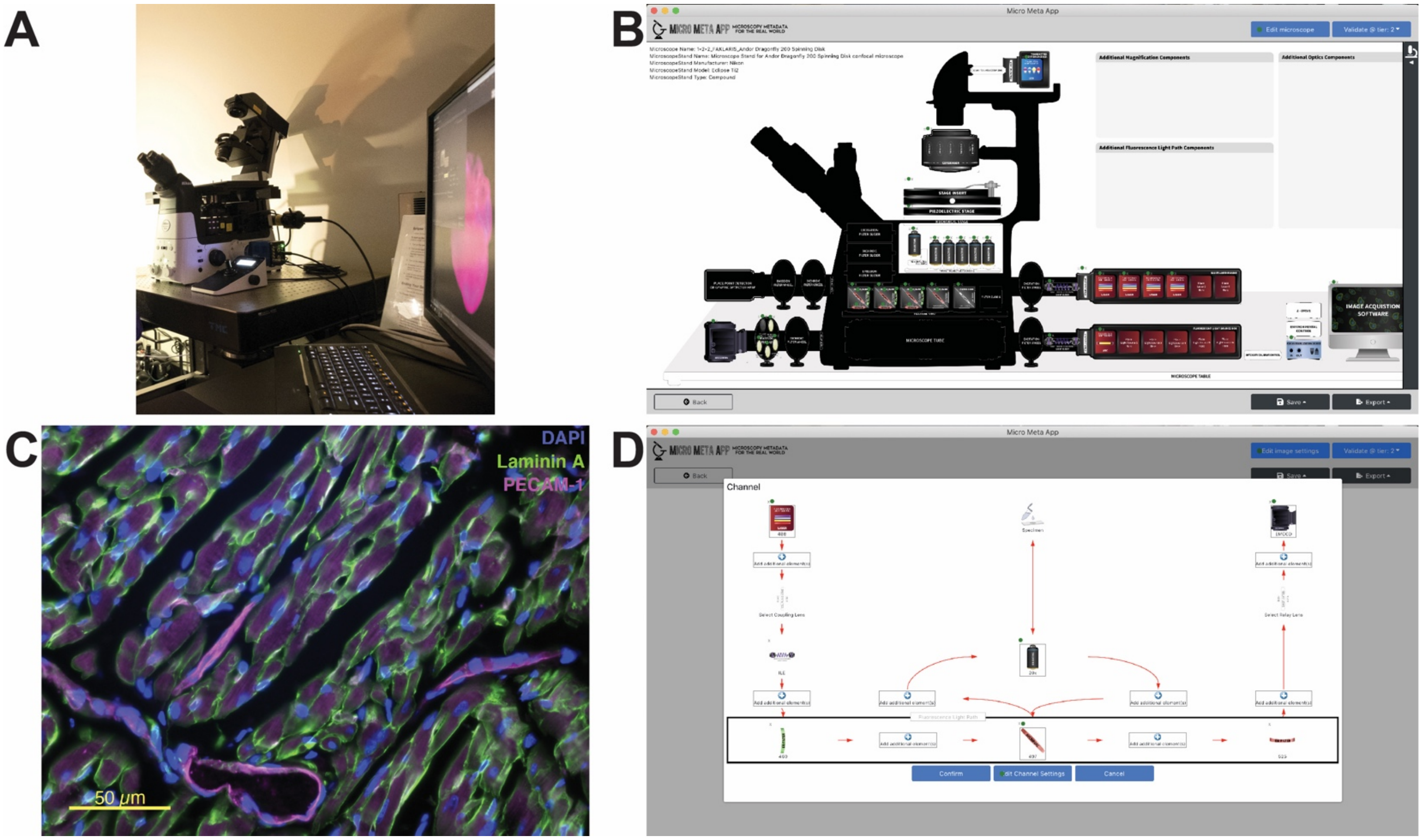

Supplementary Figure 13 | Micro-Meta App was utilized by the Microscopy Core, at the Neuroscience Center of the University of North Carolina to document a Nikon Eclipse Ti2 inverted, epifluorescence microscope and the settings that were applied to the microscope to acquire specific example datasets. Illustrated here is the use of Micro-Meta App for <u>Tier 2</u> *(Advanced Quantification and/or Live cell Imaging)*, 4DN-BINA-OME-specified[2,4] documentation of: **(A-B)** the ***Nikon Eclipse Ti2*** inverted, epifluorescence microscope owned by the Microscopy Core, at the Neuroscience Center of the University of North Carolina (Extended Data Figure 6 - Table III); and **(C-D)** the settings that were applied to the microscope for the acquisition of example published image datasets[20]. **A)** Picture of the indicated microscope. **B)** Micro-Meta App generated schematic representation of the indicated microscope. **C)** Mouse heart myocardium tissues from the left ventricles were fixed, cryo-protected with 30% sucrose overnight at 4C, embedded in OCT compound, and sectioned on a transverse plane to produce 8 µm sections. Sections were washed in PBS, permeabilized, blocked, and stained with rabbit antibodies against Laminin A (GFP Channel, visualized with Alexa Fluor 488 conjugated secondary antibody, displayed in green), rat antibodies against PECAM-1 (CD31; Texas Red Channel, visualized with Alexa Fluor 568 conjugated secondary antibody, displayed in magenta), and DAPI (displayed in blue). Displayed is a representative single plane image extracted from a 3D stack obtained from randomly selected areas of the tissue obtained using the indicated microscope (Panels A and B). The displayed image was obtained using the indicated microscope (Panels A and B)[20] **D)** Micro-Meta App generated schematic representation of the light path associated with the ***Laminin A (Alexa Fluor 488) Channel*** utilized for the acquisition of the image in Panel C. To demonstrate the functionality and usability of the App and to assess the feasibility of the overall microscopy documentation approach, Micro-Meta App was tested independently at 16 sites (Extended Data Figures 7-9 and Supplementary Figures 3-15).

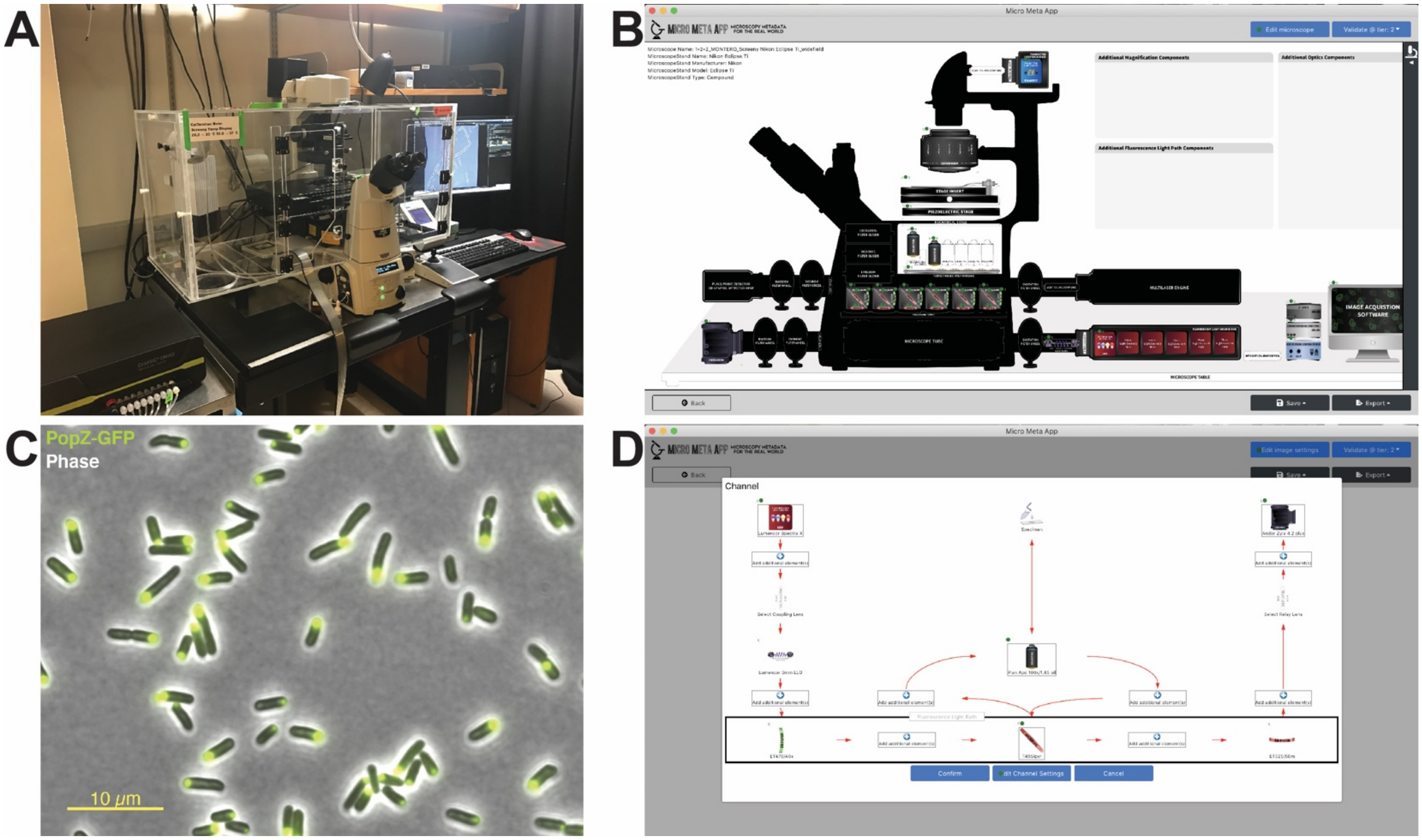

Supplementary Figure 14 | Micro-Meta App was utilized by the MicRoN Facility in the Department of Microbiology at Harvard Medical School to document a Nikon_Eclipse Ti2 inverted LED-based widefield microscope and the settings that were applied to the microscope to acquire specific example datasets. Illustrated here is the use of Micro-Meta App for Tier 2 *(Advanced Quantification and/or Live cell Imaging)*, 4DN-BINA-OME-specified[2] documentation of: **(A-B)** the ***Nikon_Eclipse Ti2*** inverted LED-based widefield microscope owned by the MicRoN Facility in the Department of Microbiology at Harvard Medical School and **(C-D)** the settings that were applied to the microscope for the acquisition of example published image datasets[23]. **A)** Picture of the indicated microscope. **B)** Micro-Meta App generated schematic representation of the indicated microscope. **C)** *Escherichia coli* strain TB28 cells expressing PopZ-superfolderGFP were grown at 37 °C in minimal M9 medium supplemented with 0.2% casamino acids and 0.2% maltose (M9 Mal medium). Prior to imaging, cultures was pelleted by gentle centrifugation. The cell pellet was resuspended and spotted on 2% agarose pads and covered with #1.5 coverslips. Overlay images show the GFP channel depicted in green, and the Phase contrast channel depicted in grey. The displayed image was obtained using the indicated microscope (Panels A and B)[23]. **D)** Micro-Meta App generated schematic representation of the light path associated with the ***PopZ-GFP Channel*** utilized for the acquisition of the image in Panel C. To demonstrate the functionality and usability of the App and to assess the feasibility of the overall microscopy documentation approach, Micro-Meta App was tested independently at 16 sites (Extended Data Figures 7-9 and Supplementary Figures 3-15).

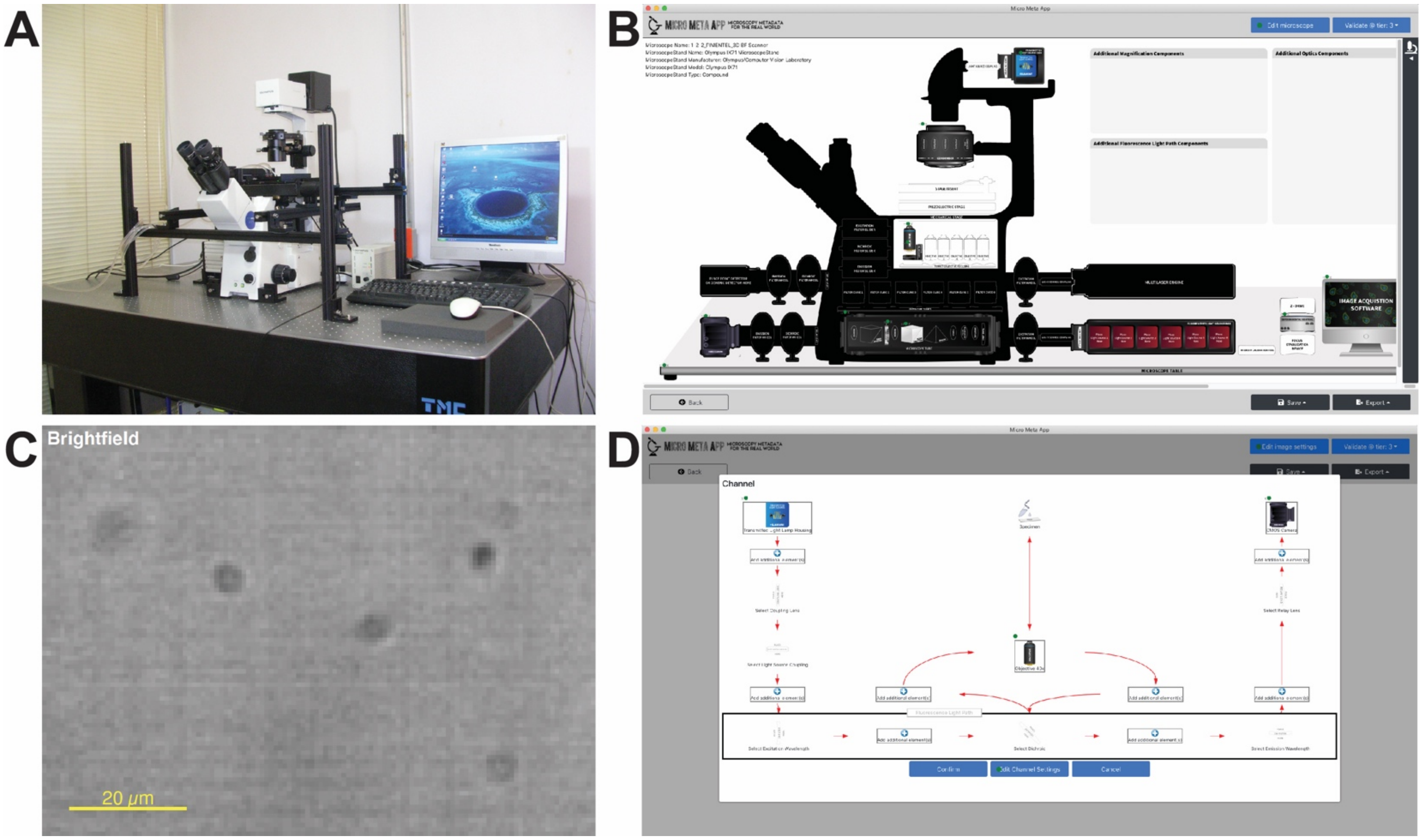

Supplementary Figure 15 | Micro-Meta App was utilized by the Computer Vision Laboratory of the Institute of Biotechnology at the Universidad Nacional Autonoma de Mexico to document a custom build 3D BrightField Scanner (based on Olympus IX71) inverted epifluorescence microscope and the settings that were applied to the microscope to acquire specific example datasets. Illustrated here is the use of Micro-Meta App for Tier 3 *(Manufacturing/Technical Development/Full Documentation)*, 4DN-BINA-OME-specified[2,4] documentation of: **(A-B)** the custom build ***3D BrightField Scanner (based on Olympus IX71)*** inverted epifluorescence microscope developed and built by the Computer Vision Laboratory of the Institute of Biotechnology and owned in conjunction with the Laboratorio Nacional de Microscopía Avanzada (LNMA) at the Universidad Nacional Autonoma de Mexico (UNAM; Extended Data Figure 6 - Table III); and **(C-D)** the settings that were applied to the microscope for the acquisition of example published image datasets[30,31]. **A)** Picture of the indicated microscope. **B)** Micro-Meta App generated schematic representation of the indicated microscope. **C)** Sperm obtained from the sea urchin *Strongylocentrotus purpuratus* were diluted in Artificial Salt Water and imaged in Petri dishes used as imaging chambers. In order to capture sperm displacement information, images were continually acquired at 2000 fps using a Optronics CR5000x2 camera during continual oscillatory 3D objective focal scanning for a 250 µm depth through the sperm sample at a frequency of 30 Hz. This acquisition rate combined with the piezoelectric movement guarantees that one frame is acquired each 8µm in the z-direction. Displayed is a single focal plane from a representative 3D+t stack. Dark spots correspond to unfocused swimming sea urchin sperm. Image obtained using the indicated microscope (Panels A and B)[30,31]. D) Micro-Meta App generated schematic representation of the light path associated with the ***Brightfield Channel*** utilized for the acquisition of the image in Panel C. To demonstrate the functionality and usability of the App and to assess the feasibility of the overall microscopy documentation approach, Micro-Meta App was tested independently at 16 sites (Extended Data Figures 7-9 and Supplementary Figures 3-15).

SUPPLEMENTARY REFERENCES

1. Hammer, M. *et al. 4DN-BINA-OME (NBO)-Microscopy Metadata Specifications - Tiers System_v2.01*. (https://github.com/WU-BIMAC/NBOMicroscopyMetadataSpecs/tree/master/Tier%20System/stable%20version/v02-01, 2021). doi: https://doi.org/10.5281/zenodo.4710731.
2. Hammer, M. *et al.* Towards community-driven metadata standards for light microscopy: tiered specifications extending the OME model - preprint. *BioRxiv* (2021) doi:10.1101/2021.04.25.441198v1.
3. Huisman, M. *et al.* A perspective on Microscopy Metadata: data provenance and quality control. *arXiv [q-bio.QM]: https://arxiv.org/abs/1910.11370* (2021).
4. Hammer, M. *et al.* Towards community-driven metadata standards for light microscopy: tiered specifications extending the OME model - Commentary. *Nature Methods (accepted after peer review)* vol. accepted after peer review (2021).
5. Rigano, A. *et al.* PREPRINT_Micro-Meta App: an interactive software tool to facilitate the collection of microscopy metadata based on community-driven specifications - preprint. *BioRxiv* (2021) doi:10.1101/2021.05.31.446382.
6. Bellve, K., Rigano, A., Fogarty, K. & Strambio-De-Castillia, C. Example Microscopy Metadata JSON files produced using Micro-Meta App to document the acquisition of example images using the custom-built TIRF Epifluorescence Structured Illumination Microscope. *Zenodo* (2021) doi:10.5281/zenodo.4891883.
7. Dekker, J. *et al.* The 4D nucleome project. *Nature* **549**, 219–226 (2017).
8. Rigano, A. *et al. Micro-Meta App – 4DN Data Portal*. (https://data.4dnucleome.org/tools/micro-meta-app, 2021). doi:10.5281/zenodo.5140157.
9. Ryan, J. *et al.* MethodsJ2: A Software Tool to Improve Microscopy Methods Reporting - preprint. *BioRxiv* (2021) doi:10.1101/2021.06.23.449674.
10. Ryan, J. *et al.* MethodsJ2: A Software Tool to Capture and Generate Comprehensive Microscopy Methods Text and Improve Reproducibility. *Nat. Methods* **in press**, (2021).
11. Linkert, M. *et al.* Metadata matters: access to image data in the real world. *J. Cell Biol.* **189**, 777–782 (2010).
12. Alver, B., 4DN-DCIC & Park, P. 4DN Data Portal. *https://data.4dnucleome.org/* https://data.4dnucleome.org/ (2018).
13. Abdelfattah, A. S. *et al.* Bright and photostable chemigenetic indicators for extended in vivo voltage imaging. *Science* **365**, 699–704 (2019).
14. Qian, Y. *et al.* A genetically encoded near-infrared fluorescent calcium ion indicator. *Nat. Methods* **16**, 171–174 (2019).
15. Grimm, J. B. *et al.* A general method to optimize and functionalize red-shifted rhodamine dyes. *Nat. Methods* **17**, 815–821 (2020).
16. Kiepas, A., Voorand, E., Mubaid, F., Siegel, P. M. & Brown, C. M. Optimizing live-cell fluorescence imaging conditions to minimize phototoxicity. *J. Cell Sci.* **133**, (2020).
17. Fernandez, A. *et al.* Programmable Emulsions via Nucleophile-Induced Covalent Surfactant Modifications. *Chem. Mater.* **32**, 4663–4671 (2020).
18. Drosopoulos, W. C., Vierra, D. A., Kenworthy, C. A., Coleman, R. A. & Schildkraut, C. L. Dynamic Assembly and Disassembly of the Human DNA Polymerase δ Holoenzyme on the Genome In Vivo. *Cell Rep.* **30**, 1329–1341.e5 (2020).
19. Ayala-Nunez, N. V. *et al.* Zika virus enhances monocyte adhesion and transmigration favoring viral dissemination to neural cells. *Nat. Commun.* **10**, 4430 (2019).
20. Aghajanian, A. *et al.* Decreased inspired oxygen stimulates de novo formation of coronary collaterals in adult heart. *J. Mol. Cell. Cardiol.* **150**, 1–11 (2021).
21. Watson, N. A. *et al.* Kinase inhibition profiles as a tool to identify kinases for specific phosphorylation sites. *Nat. Commun.* **11**, 1684 (2020).
22. Upton, R. L., Davies-Manifold, Z., Marcello, M., Arnold, K. & Crick, C. R. A general formulation approach for the fabrication of water repellent materials: how composition can impact resilience and functionality. *Mol. Syst. Des. Eng.* **5**, 477–483 (2020).
23. Lim, H. C. & Bernhardt, T. G. A PopZ-linked apical recruitment assay for studying protein-protein interactions in the bacterial cell envelope. *Mol. Microbiol.* **112**, 1757–1768 (2019).
24. Lim, H. C. *et al.* Identification of new components of the RipC-FtsEX cell separation pathway of Corynebacterineae. *PLoS Genet.* **15**, e1008284 (2019).

25. da Silva, P. F. L. *et al.* The bystander effect contributes to the accumulation of senescent cells in vivo. *Aging Cell* **18**, e12848 (2019).
26. Dalle Pezze, P. *et al.* Dynamic Modelling of Pathways to Cellular Senescence Reveals Strategies for Targeted Interventions. *PLoS Comput. Biol.* **10**, 1–20 (2014).
27. Proksch, S. *et al.* hMSC-Derived VEGF Release Triggers the Chemoattraction of Alveolar Osteoblasts. *Stem Cells* **33**, 3114–3124 (2015).
28. Navaroli, D. M. *et al.* Rabenosyn-5 defines the fate of the transferrin receptor following clathrin-mediated endocytosis. *Proceedings of the National Academy of Sciences* **109**, E471–80 (2012).
29. Hannibal, L. *et al.* Metabolic Profiling in Human Fibroblasts Enables Subtype Clustering in Glycogen Storage Disease. *Front. Endocrinol.* **11**, 579981 (2020).
30. Pimentel, J. A., Carneiro, J., Darszon, A. & Corkidi, G. A segmentation algorithm for automated tracking of fast swimming unlabelled cells in three dimensions. *J. Microsc.* **245**, 72–81 (2012).
31. Silva-Villalobos, F., Pimentel, J. A., Darszon, A. & Corkidi, G. Imaging of the 3D dynamics of flagellar beating in human sperm. *Conf. Proc. IEEE Eng. Med. Biol. Soc.* **2014**, 190–193 (2014).
32. Jubrail, J. *et al.* Arpin is critical for phagocytosis in macrophages and is targeted by human rhinovirus. *EMBO Rep.* **21**, e47963 (2020).
33. Rigano, A. *et al. 4DN-BINA-OME (NBO) Tiered Microscopy Metadata Specifications - v2.01*. (https://github.com/WU-BIMAC/NBOMicroscopyMetadataSpecs, 2021). doi:10.5281/zenodo.4710731.
34. Singer, A. *et al.* Massive Protein Import into the Early-Evolutionary-Stage Photosynthetic Organelle of the Amoeba Paulinella chromatophora. *Curr. Biol.* **27**, 2763–2773.e5 (2017).
35. Hammer, M. & Grunwald, D. Foundations in Biomedical Science (BBS 614) graduate course. *Courses of the Graduate School of Biomedical Sciences* https://www.umassmed.edu/gsbs/academics/courses/ (2020).
36. Smith, C. S. *et al.* Nuclear accessibility of β-actin mRNA is measured by 3D single-molecule real-time tracking. *J. Cell Biol.* **209**, 609–619 (2015).
37. Pilliod, J. *et al.* Clearance of intracellular tau protein from neuronal cells via VAMP8-induced secretion. *J. Biol. Chem.* **295**, 17827–17841 (2020).
38. Nelson, G. *et al.* A senescent cell bystander effect: senescence-induced senescence. *Aging Cell* **11**, 345–349 (2012).
