# Supplementary material for: Micro-Meta App: an interactive tool for collecting microscopy metadata based on community specifications
Source: Nat Methods. 2021 Dec 3;18(12):1489–95. doi: 10.1038/s41592-021-01315-z (PMC8648560; doi:10.1038/s41592-021-01315-z)
Supplement: Supplementary file 2 — Reporting Summary [file 41592_2021_1315_MOESM2_ESM.pdf]

nature research

Corresponding author(s): Caterina Strambio-De-Castillia

Last updated by author(s): 2021-9-14

Reporting Summary

Nature Research wishes to improve the reproducibility of the work that we publish. This form provides structure for consistency and transparency in reporting. For further information on Nature Research policies, see our Editorial Policies and the Editorial Policy Checklist.

Statistics

For all statistical analyses, confirm that the following items are present in the figure legend, table legend, main text, or Methods section.

n/a	Confirmed	
☐	☒	The exact sample size (n) for each experimental group/condition, given as a discrete number and unit of measurement
☒	☐	A statement on whether measurements were taken from distinct samples or whether the same sample was measured repeatedly
☒	☐	The statistical test(s) used AND whether they are one- or two-sided<br>*Only common tests should be described solely by name; describe more complex techniques in the Methods section.*
☒	☐	A description of all covariates tested
☒	☐	A description of any assumptions or corrections, such as tests of normality and adjustment for multiple comparisons
☒	☐	A full description of the statistical parameters including central tendency (e.g. means) or other basic estimates (e.g. regression coefficient) AND variation (e.g. standard deviation) or associated estimates of uncertainty (e.g. confidence intervals)
☒	☐	For null hypothesis testing, the test statistic (e.g. F, t, r) with confidence intervals, effect sizes, degrees of freedom and P value noted<br>*Give P values as exact values whenever suitable.*
☒	☐	For Bayesian analysis, information on the choice of priors and Markov chain Monte Carlo settings
☒	☐	For hierarchical and complex designs, identification of the appropriate level for tests and full reporting of outcomes
☒	☐	Estimates of effect sizes (e.g. Cohen's d, Pearson's r), indicating how they were calculated

Our web collection on statistics for biologists contains articles on many of the points above.

Software and code

Policy information about availability of computer code

Data collection

This manuscript describes the Micro-Meta App software tool for Microscopy Metadata collection to document the hardware specifications and image acquisition settings utilized to produce microscopy image data and using fluorescence microscopes.
As listed in the "Availability, requirements, resources, and documentation" section of the Online Methods, and on the README file on GiitHub (https://github.com/WU-BIMAC/MicroMetaApp-Electron) the software is available as follows:

● Project name: Micro-Meta App
● Project home page: https://github.com/WU-BIMAC/MicroMetaApp.github.io
● Documentation (including video tutorials): https://micrometaapp-docs.readthedocs.io/en/latest/index.html
○ Note: if you intend to use the Micro-Meta App on MacOS you might encounter difficulties un-zipping and launching the MacOS Zip. To address these issues please follow the special instructions specified in this VIDEO → https://vimeo.com/529609242
● Downloadable stand-alone executable available at:
○ Desktop application (Javascript Electron): https://github.com/WU-BIMAC/MicroMetaApp-Electron/releases/latest (DOI: https://doi.org/10.5281/zenodo.4750765)
● Source code and README with instructions available at:
○ Desktop application (Javascript Electron): https://github.com/WU-BIMAC/MicroMetaApp-Electron (DOI: https://doi.org/10.5281/zenodo.4750765)
○ Data-portal application (Javascript React): https://github.com/WU-BIMAC/MicroMetaApp-React (DOI: https://doi.org/10.5281/zenodo.4889259)
● Example Dataset:
○ Available at: https://doi.org/10.5281/zenodo.4891883
○ Content:
-- Microscope.JSON
-- Settings.JSON
-- associated raw image data file (Figure 2 and Extended Data Figure 9 use case)

-- links to instructional video tutorials
● Operating system(s):
○ Windows x32
○ Windows x64
Tested on:
-- Win 8.1 Version: 6.3.9600
-- Win 10 Home Version: 10.0.19040 - 10.0.19041 - 10.0.19042
-- Win 10 Pro Version: 10.0.19041 - 10.0.19042
-- Win 10 Enterprise Version: 10.0.18362, 10.0.19041, 10.0.19042
○ MacOS
Tested on:
-- High Sierra Version: 10.13.6
-- Mojave Version: 10.14.6
-- Catalina Verson: 10.15.7
-- Big Sur Version: 11.0 - 11.1 - 11.2 - 11.3 - 11.3.1 - 11.4 - 11.5 Beta
● Programming language: Javascript and Java
● Other requirements: Java v1.8.0. In addition, see dependencies listed in Supplemental Material.
● License: GNU GPL v3 (https://www.gnu.org/licenses/gpl-3.0.html)

Data analysis

No data analysis was performed for this study.

For manuscripts utilizing custom algorithms or software that are central to the research but not yet described in published literature, software must be made available to editors and reviewers. We strongly encourage code deposition in a community repository (e.g. GitHub). See the Nature Research guidelines for submitting code & software for further information.

Data

Policy information about availability of data

All manuscripts must include a data availability statement. This statement should provide the following information, where applicable:

- Accession codes, unique identifiers, or web links for publicly available datasets
- A list of figures that have associated raw data
- A description of any restrictions on data availability

• Example data-files associated with Figure 2, and Extended Data Figure 9 and utilized at UMass Medical School demonstrate the usability of Micro-Meta App to document microscopy experiments are publicly available on Zenodo as follows:
○ Title: "Example Microscopy Metadata JSON files produced using Micro-Meta App to document the acquisition of example images using the custom-built TIRF Epifluorescence Structured Illumination Microscope"
○ Available at: https://doi.org/10.5281/zenodo.4891883
○ Content:
-- Microscope.JSON
-- Settings.JSON
-- associated raw image data file (Figure 2 and Extended Data Figure 9 use case)
-- links to instructional video tutorials

• Example datasets associated with Extended Data Figures 7-8 and Supplemental Figures 3-15 and utilized at 16 different imaging core facilities to evaluate the functionality and test the usability of Micro-Meta App, can be made available upon request from the corresponding author. This include exemplar image data files that were not produced to test hypotheses or reach conclusions that are part of this study but were successfully utilized as case studies to test the feasibility of the Micro-Meta App approach.

• Data associated with Extended Data Figure 10 is available publicly on the 4DN Data Portal as follows:
Panel A) https://data.4dnucleome.org/files-microscopy/4DNFI7639BEB/;
Panel B) https://omero.hms.harvard.edu/pathviewer/vanilla-viewer/975042/;
Panel C) https://data.4dnucleome.org/microscope-configurations/28f1c0f2-d903-4761-93c6-dd3994db3462/.

• Supplemental Video 1 is also available publicly at: https://vimeo.com/manage/videos/604291798

Field-specific reporting

Please select the one below that is the best fit for your research. If you are not sure, read the appropriate sections before making your selection.

☒ Life sciences ☐ Behavioural & social sciences ☐ Ecological, evolutionary & environmental sciences

For a reference copy of the document with all sections, see nature.com/documents/nr-reporting-summary-flat.pdf

Life sciences study design

All studies must disclose on these points even when the disclosure is negative.

Sample size

Micro-Meta App was utilized in 16 separate international imaging core facilities to collect the hardware specifications and image acquisition settings utilized to produce example microscopy image data and using representative fluorescence microscopes.
16 independent sites were selected for independent testing because they reflected multiple different microscope modalities and experiment

sites.
Because software usage at all sites was conducted successfully as indicated in the Main Text and in Supplemental Material, the testing size was deemed sufficient to evaluate the feasibility of the Micro-Meta App approach.

Data exclusions	none was performed
Replication	The images presented in the Case Studies figures (Extended Data Figure 7-9 and Supplemental Figure 3-15) and as listed in Extended Data Figure 6 - Table III are presented in this study only as example of possible use of Micro-Meta App and as exemplars of the type of imaging experiments that can be documented by Micro-Meta App. As such the images do not per represent the results of experiments whose conclusions is relevant to this study.<br>More specifically, as part of this study no biological hypothesis were tested, or conclusions were drawn from these images that would warrant performing a statistical analysis or the reporting of repetition or sample size.<br><br>Instead, the result of this study consists in the ability of members of the different testing sites to use Micro-Meta App to document the Hardware Specifications of the described microscopes as well as the image Acquisition Settings that were used for the raw images displayed in each of the listed Figures.<br><br>As such, the relevant number of repetitions to evaluate the REPRODUCIBLITY of this study consists in the number of times Micro-Meta App was tested in parallel and independently at different testing sites. This number is 16 as indicated and justified under section "Sample Size" in this document and in the Main Text under section: "Case studies: utilization at core facilities".<br><br>Based on this reasoning the following sentence was added to the legend of all relevant figures: "To demonstrate the functionality and usability of the App and to assess the feasibility of the overall approach to document the provenance and quality control of microscopy experiments, Micro-Meta App was tested independently at 16 sites."
Randomization	The images presented in the Case Studies figures (Extended Data Figure 7-9 and Supplemental Figure 3-15) and as listed in Extended Data Figure 6 - Table III are presented in this study only as example of possible use of Micro-Meta App and as exemplars of the type of imaging experiments that can be documented by Micro-Meta App.<br>As such the images do not per represent the results of experiments whose conclusions is relevant to this study.<br>More specifically, as part of this study no biological hypothesis were tested, or conclusions were drawn from these images that would warrant performing a randomization.
Blinding	The images presented in the Case Studies figures (Extended Data Figure 7-9 and Supplemental Figure 3-15) and as listed in Extended Data Figure 6 - Table III are presented in this study only as example of possible use of Micro-Meta App and as exemplars of the type of imaging experiments that can be documented by Micro-Meta App.<br>As such the images do not per represent the results of experiments whose conclusions is relevant to this study.<br>More specifically, as part of this study no biological hypothesis were tested, or conclusions were drawn from these images that would warrant performing a binding.

Reporting for specific materials, systems and methods

We require information from authors about some types of materials, experimental systems and methods used in many studies. Here, indicate whether each material, system or method listed is relevant to your study. If you are not sure if a list item applies to your research, read the appropriate section before selecting a response.

Materials & experimental systems

n/a	Involved in the study
☒	☐ Antibodies
☒	☐ Eukaryotic cell lines
☒	☐ Palaeontology and archaeology
☒	☐ Animals and other organisms
☒	☐ Human research participants
☒	☐ Clinical data
☒	☐ Dual use research of concern

Methods

n/a	Involved in the study
☒	☐ ChIP-seq
☒	☐ Flow cytometry
☒	☐ MRI-based neuroimaging
